# The Nuclear Receptor HIZR-1 Uses Zinc as a Ligand to Mediate Homeostasis in Response to High Zinc

**DOI:** 10.1371/journal.pbio.2000094

**Published:** 2017-01-17

**Authors:** Kurt Warnhoff, Hyun C. Roh, Zuzana Kocsisova, Chieh-Hsiang Tan, Andrew Morrison, Damari Croswell, Daniel L. Schneider, Kerry Kornfeld

**Affiliations:** Department of Developmental Biology, Washington University School of Medicine, St. Louis, Missouri, United States of America; Brandeis University, United States of America; University of Massachusetts Medical School, United States of America

## Abstract

Nuclear receptors were originally defined as endocrine sensors in humans, leading to the identification of the nuclear receptor superfamily. Despite intensive efforts, most nuclear receptors have no known ligand, suggesting new ligand classes remain to be discovered. Furthermore, nuclear receptors are encoded in the genomes of primitive organisms that lack endocrine signaling, suggesting the primordial function may have been environmental sensing. Here we describe a novel *Caenorhabditis elegans* nuclear receptor, HIZR-1, that is a high zinc sensor in an animal and the master regulator of high zinc homeostasis. The essential micronutrient zinc acts as a HIZR-1 ligand, and activated HIZR-1 increases transcription of genes that promote zinc efflux and storage. The results identify zinc as the first inorganic molecule to function as a physiological ligand for a nuclear receptor and direct environmental sensing as a novel function of nuclear receptors.

## Author Summary

Zinc is an essential nutrient for all life forms, and maintaining zinc homeostasis is critical for survival. However, little is known about how animals sense changes in zinc availability and make adjustments to maintain homeostasis. In particular, logic dictates there must be a mechanism for zinc sensing, but it has not been defined in animals. We discovered that the nuclear receptor transcription factor HIZR-1 is the master regulator of high zinc homeostasis in the roundworm *Caenorhabditis elegans*. In response to high dietary zinc, HIZR-1 activates transcription of multiple genes that encode a network of proteins that store and detoxify excess zinc. Furthermore, our results suggest HIZR-1 itself is the high zinc sensor, since it directly binds zinc ions in the ligand-binding domain that regulates transcriptional activation. These findings advance the understanding of zinc homeostasis and nuclear receptor biology. Nuclear receptors were initially characterized as receptors for hormones such as estrogen, indicating complex animals use these transcription factors to monitor their internal environment. However, nuclear receptors are present in simple organisms that lack endocrine signaling, suggesting these transcription factors might have a primordial function in sensing the external environment. Our results identify a new class of nuclear receptor ligands, the inorganic ion zinc, and a new function for nuclear receptors in directly sensing levels of a nutrient. We speculate that nutrient homeostasis mediated by direct binding of nutrients to the ligand-binding domain is a primordial function of nuclear receptors, whereas endocrine signaling in complex animals mediated by direct binding of hormones to the ligand-binding domain is a derived function of nuclear receptors that appeared later in evolution.

## Introduction

Zinc is an essential nutrient for all life, including plants, animals and microbes, because zinc is involved in many different cellular events. Zn^2+^ binds tightly to many proteins and thereby contributes to their tertiary structure or catalytic activity [1], and Zn^2+^ has been proposed to function as a second messenger signaling molecule during synaptic transmission, development, and immune responses [2–4]. Zinc homeostasis is vital for human health. Inadequate dietary intake is a prevalent cause of human zinc deficiency, whereas genetic disorders that disrupt zinc uptake occur rarely. Zinc deficiency causes pathological changes in a wide range of tissues, reflecting the many uses of zinc [5–7]. Zinc excess also causes human pathology, and it may occur systemically or in specific tissues. For example, ischemic injury has been proposed to cause zinc release that mediates cell death [8,9]. Several human diseases, including diabetes, cancer, and neurodegenerative diseases, are correlated with genetic variations that affect zinc metabolism [10–12]. Elucidating zinc homeostasis is important for understanding an ancient biological process and may contribute to improving human health.

Because excess zinc is toxic, organisms require mechanisms to sense and detoxify high levels of zinc. One fundamental and evolutionarily conserved mechanism is transcriptional regulation of genes involved in zinc detoxification, such as zinc exporters and zinc sequestering proteins [13–16]. However, the regulation of high-zinc–activated transcription remains poorly understood in animals. Critical questions in this field include the following: What is the direct sensor of high zinc? And how does this sensor activate transcription of zinc homeostasis genes?

The nematode *Caenorhabditis elegans* is a useful model system for studies of zinc homeostasis because its simple body plan, its transparency, and the availability of powerful genetic techniques facilitate experimental analysis [13,17–21]. Studies of *C*. *elegans* are relevant to mammalian biology, since the genome encodes evolutionarily conserved zinc transporters and metallothioneins [22,23]. The transcription of these genes is regulated by dietary zinc levels in *C*. *elegans*, which is also similar to yeast and mammals [24,25]. *C*. *elegans* contains two metallothionein genes, *mtl-1* and *mtl-2*, that are induced at the level of transcription in intestinal cells by high dietary zinc [26]. *C*. *elegans* contains 14 cation diffusion facilitator (CDF) genes that encode transporters for zinc and possibly other metal ions. The CDF genes *cdf-2* and *ttm-1b* are transcriptionally up-regulated in intestinal cells by high dietary zinc, and these transporters promote zinc storage and excretion [14,19]. Transcriptional activation of these genes is mediated by the high zinc activation (HZA) element, a DNA enhancer [15]. However, the HZA-binding factor has not been identified.

Here we describe an unbiased forward genetic screen used to identify mediators of high-zinc–activated transcription that resulted in the discovery of the HZA-binding factor, which we named the *hi*gh-*z*inc–activated nuclear *r*eceptor (HIZR-1). *hizr-1* encodes a nuclear receptor transcription factor that has an evolutionarily conserved DNA-binding domain (DBD) and ligand-binding domain (LBD). We demonstrated that HIZR-1 is both necessary and sufficient to activate transcription of endogenous zinc-homeostasis genes in response to high dietary zinc. Thus, HIZR-1 is the master regulator of high-zinc homeostasis in *C*. *elegans*. We used genetic and biochemical approaches to analyze HIZR-1 function. The LBD directly bound zinc, which promoted nuclear accumulation and activation of the protein, indicating the LBD regulates protein activity and zinc is a physiological ligand; the DBD directly bound the HZA enhancer, which mediates transcriptional activation of multiple genes involved in zinc homeostasis [15]. These findings advance the understanding of zinc biology by identifying a sensor for high zinc in animals and elucidate homeostatic systems by defining a positive feedback loop embedded in a negative feedback circuit.

Nuclear receptors (NRs) were discovered and characterized as sensors of endocrine signals in mammals. These transcription factors contain a regulatory ligand-binding domain that interacts with hormones and a DNA-binding domain that interacts with target genes. Despite decades of effort, only about half of human NRs have identified ligands, raising the possibility that novel classes of ligands remain to be discovered [27]. NRs exist in simple organisms such as sponges that lack endocrine signaling, suggesting NRs might have primordial function in sensing external molecules [28]. However, external ligands have yet to be identified. The analysis of HIZR-1 expands the understanding of the NR superfamily by (1) identifying transition metals as a new class of physiological ligand that is distinct from previously described classes such as steroids and lipids and (2) identifying direct nutrient sensing as a new function that may represent a primordial role of NRs.

## Results

### A Genetic Screen for Abnormal High-Zinc–Activated Transcription

**Fig 1:**
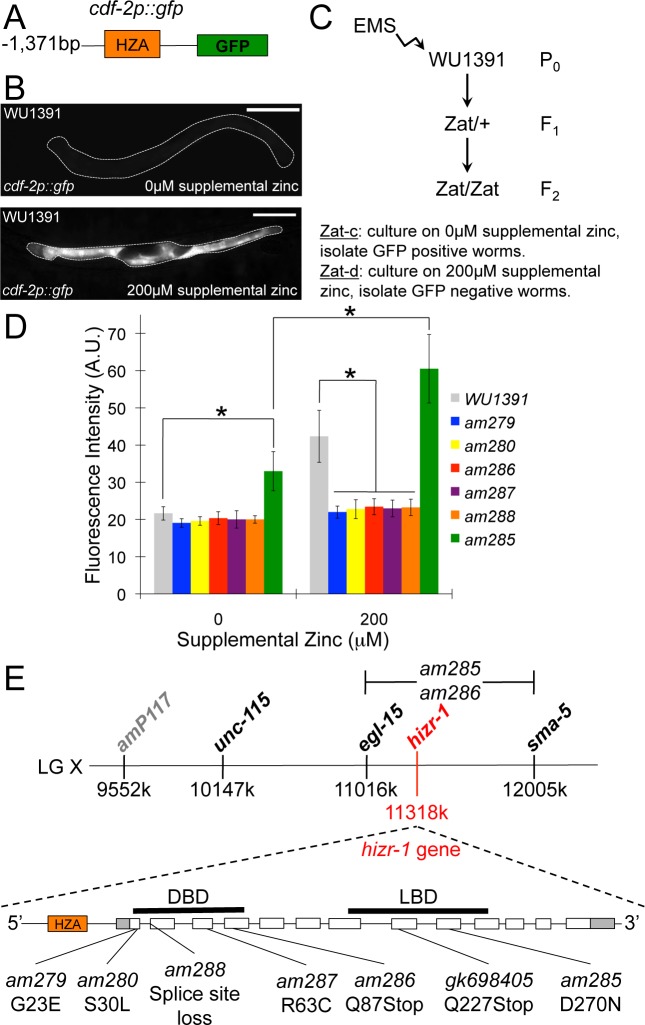
A forward genetic screen for mutations that affect zinc-activated transcription of the *cdf-2* promoter. **(A)** A diagram (not to scale) of the *cdf-2p*::*gfp* transcriptional reporter construct containing the *cdf-2* promoter (black line, 1,371bp upstream of the ATG start codon) fused to the coding region of green fluorescence protein (green box). The *cdf-2* promoter contains a high zinc activation (HZA) enhancer element (orange box, 194 bp upstream of the ATG start codon) [15]. This construct was integrated into the genome of *C*. *elegans* to generate transgenic strain WU1391 that functions as a high zinc reporter strain. **(B)** WU1391 transgenic animals containing the *cdf-2p*::*gfp* transcriptional reporter were cultured with standard Noble agar minimal medium (NAMM) (0 μM Supplemental Zinc) or NAMM supplemented with 200 μM supplemental zinc. GFP fluorescence was observed with a compound microscope, and representative images are shown. Animals were oriented with the long axis horizontally—the intestine is a prominent tubular structure spanning the length of the animal, and it is outlined with a dotted white line. Animals cultured in medium with no supplemental zinc displayed low-level fluorescence that is a combination of autofluorescence due to gut granules and low level expression of the *cdf-2* promoter. By contrast, intestinal fluorescence is prominent in animals cultured in zinc-supplemented medium. Scale bars are approximately 100 μm. **(C)** A flow chart of the forward genetic screen. WU1391 transgenic hermaphrodites (P_0_) were mutagenized by ethyl methanesulfonate (EMS) and allowed to self-fertilize for two generations. F_2_ self progeny that are homozygous for newly induced mutations were analyzed. *Z*inc-*a*ctivated *t*ranscription-*c*onstitutive (Zat-c) mutants were isolated by screening for animals that displayed fluorescence when cultured on medium with no supplemental zinc. *Z*inc-*a*ctivated *t*ranscription-*d*eficient (Zat-d) mutants were isolated by screening for animals that did not display fluorescence when cultured on medium with 200 μM supplemental zinc. **(D)** WU1391, the Zat-d mutant strains (*am279*, *am280*, *am286*, *am287*, and *am288*) and the Zat-c mutant strain (*am285*) were cultured on medium with 0 or 200 μM supplemental zinc. Fluorescence intensity was quantified using microscopy and is expressed in arbitrary units (A.U.). Bars represent the average fluorescence intensity +/- standard deviation (S.D.) (*n* = 12–18 animals). Compared to the unmutagenized WU1391 starting strain, all five Zat-d mutant strains displayed significantly reduced fluorescence intensity when cultured with 200 μM supplemental zinc; the one Zat-c mutant strain displayed significantly increased fluorescence intensity when cultured with 0 μM supplemental zinc (*, *p* < 0.05). The Zat-c mutant strain cultured on 200 μM supplemental zinc displayed significantly increased levels of GFP fluorescence compared to the Zat-c mutant strain cultured on 0 μM supplemental zinc and WU1391 animals cultured on 200 μM supplemental zinc. Thus, the Zat-c mutant strain retains zinc-activated transcription while displaying higher baseline expression levels compared to wild type. **(E)** Upper, physical map of a portion of *C*. *elegans* linkage group X (LGX) with loci positions in base pairs (k = thousand). Zat-d and Zat-c mutations were positioned between *egl-15* and *sma-5*. Lower, *hizr-1* locus (not to scale). Open boxes are exons, shaded regions are untranslated, orange box is the HZA enhancer, black bars locate the DNA-binding (DBD) and ligand-binding (LBD) domains, and lines locate mutations.

Homeostasis in response to high zinc is mediated by transcriptional activation of multiple genes in *C*. *elegans*, including the zinc transporter genes *cdf-2 and ttm-1b* [13,14]. To identify genes that mediate this response, we screened for mutant animals that displayed abnormal regulation of *cdf-2*. To visualize *cdf-2* transcription, we used the method of bombardment to generate transgenic animals with an integrated, multicopy array containing a plasmid with the *cdf-2* promoter fused to the coding region for green fluorescent protein (GFP) (*cdf-2p*::*gfp*) (see Materials and Methods). Transgenic *cdf-2p*::*gfp* animals displayed low-level fluorescence in standard medium and high-level fluorescence in intestinal cells in medium supplemented with zinc (**Fig 1A and 1B**). We identified one semidominant mutation (*am285*) that caused increased fluorescence in standard medium, a phenotype we named *z*inc-*a*ctivated *t*ranscription-*c*onstitutive (Zat-c). We identified five recessive mutations (*am279*, *am280*, *am286*, *am287*, and *am288*) that caused reduced fluorescence in medium supplemented with zinc, a phenotype we named *z*inc-*a*ctivated *t*ranscription-*d*eficient (Zat-d) (**Fig 1C and 1D**). All six mutant strains displayed growth rates similar to wild type when cultured on standard medium. All five Zat-d mutations failed to complement one another, indicating they affect the same gene. The Zat-c and Zat-d complementation groups were positioned in the center of linkage group X (**Fig 1E**).

**Fig 2:**
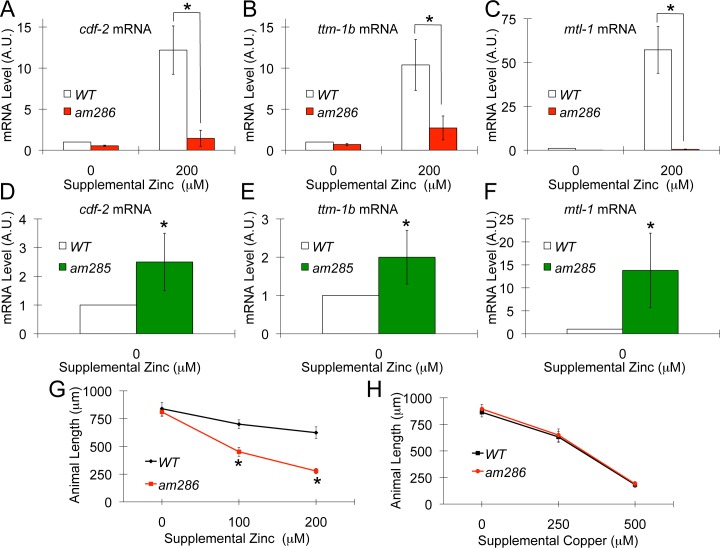
*hizr-1* was necessary and sufficient for high-zinc–activated transcription. **(A–F)** mRNA was isolated from populations of wild-type (white), *am286* (red), or *am285* (green) animals cultured with 0 or 200 μM supplemental zinc. *cdf-2*, *ttm-1b*, and *mtl-1* transcript levels were analyzed by qPCR; mRNA levels are expressed in arbitrary units (A.U.) and were normalized to *rps-23*, a ribosomal protein gene that is not regulated by high zinc. For each panel, the values were normalized by setting the value for wild-type animals at 0 μM supplemental zinc equal to 1.0. Bars represent the average +/- S.D. (*n* = 3), (*, *p* < 0.05). **(A–C)** In wild-type animals, *cdf-2*, *ttm-1b*, and *mtl-1* transcript levels were increased significantly when cultured with 200 μM supplemental zinc compared to 0 μM supplemental zinc, demonstrating that these are high-zinc–activated transcripts. Compared to wild-type animals, *am286* mutant animals displayed significantly lower mRNA levels when cultured with 200 μM supplemental zinc, indicating a defect of high-zinc–activated transcription. *cdf-2*, *ttm-1b*, and *mtl-1* mRNA levels displayed a small but statistically significant reduction in *am286* mutant animals cultured on 0 μM supplemental zinc compared to wild type cultured on 0 μM supplemental zinc or *am286* mutant animals cultured on 200 μM supplemental zinc. **(D–F)** Compared to wild-type animals, *am285* mutant animals displayed significantly higher mRNA levels when cultured with 0μM supplemental zinc, indicating constitutive activation of high-zinc–activated transcription. **(G,H)** Animals were synchronized as embryos and cultured with the indicated concentrations of supplemental zinc or copper for 3 d. Data points represent average animal length +/- S.D. (*n* = 20) (*, *p* < 0.05).

To determine how these mutations affect transcription of endogenous genes, we analyzed mRNA levels by quantitative PCR (qPCR). In wild-type animals, mRNA levels of *cdf-2*, *ttm-1b* and the metal binding metallothionein, *mtl-1*, are increased significantly by zinc supplementation [14,19]. By contrast, in *am286* mutant animals *cdf-2*, *ttm-1b*, and *mtl-1* transcript levels were significantly lower than wild type when exposed to supplemental zinc (**Fig 2A–2C**). Furthermore, the strain with the *am285* semidominant mutation displayed significantly higher levels of *cdf-2*, *ttm-1b*, and *mtl-1* transcripts compared to wild type in the absence of supplemental zinc (**Fig 2D–2F**). Thus, the gene affected by *am286* was necessary and the gene affected by *am285* was sufficient for high-zinc–activated transcription of multiple endogenous genes.

To test the hypothesis that the gene affected by *am286* is functionally important for zinc homeostasis, we analyzed growth in the presence of supplemental zinc. *am286* mutant animals grew similarly to wild-type animals in standard medium, demonstrating the strain is healthy in standard conditions, but displayed retarded growth compared to wild-type animals when cultured in high dietary zinc (**Fig 2G**). This growth defect was specific to zinc toxicity, since *am286* mutant animals displayed growth similar to wild-type animals in high dietary copper (**Fig 2H**). Thus, the gene affected by *am286* was necessary for normal growth and development in high dietary zinc.

### The Nuclear Receptor Gene *hizr-1* Was Necessary and Sufficient to Promote High-Zinc–Activated Transcription

To identify the affected gene, we performed whole genome sequencing. All six mutant strains contained mutations in the gene *ZK455*.*6/nhr-33*, which is in the mapping interval. A comparison of the predicted protein sequence to protein databases revealed homology to nuclear receptors, and we named the gene *hi*gh-*z*inc–activated nuclear *r*eceptor (*hizr-1*) (**S1 Fig**). HIZR-1 has a conserved DBD that contains two predicted zinc-finger DNA-binding motifs, which is typical of nuclear receptors, and a conserved LBD. The recessive Zat-d alleles include three encoding substitutions of conserved residues in the DBD and two encoding truncated proteins (one nonsense and one change in a consensus splicing site). The semidominant, Zat-c allele encodes a substitution affecting the LBD (**Fig 1E, S1 Table**). To confirm this gene assignment, we analyzed an independently derived allele and performed rescue experiments. *hizr-1(gk698405)*, a nonsense mutation generated by the *C*. *elegans* million mutations project [29], caused a Zat-d phenotype, as predicted (**Fig 1E, S2A Fig, S1 Table**). Expression of wild-type HIZR-1 protein fused to GFP rescued the *am286* Zat-d mutant phenotype, as predicted (**S2B and S2C Fig**). The molecular and genetic analyses indicate that the five Zat-d mutations are likely strong loss-of-function or null alleles of *hizr-1*, whereas the Zat-c mutation is likely a gain-of-function allele of *hizr-1*.

### Zinc Directly Bound the Ligand-Binding Domain of HIZR-1

**Fig 3:**
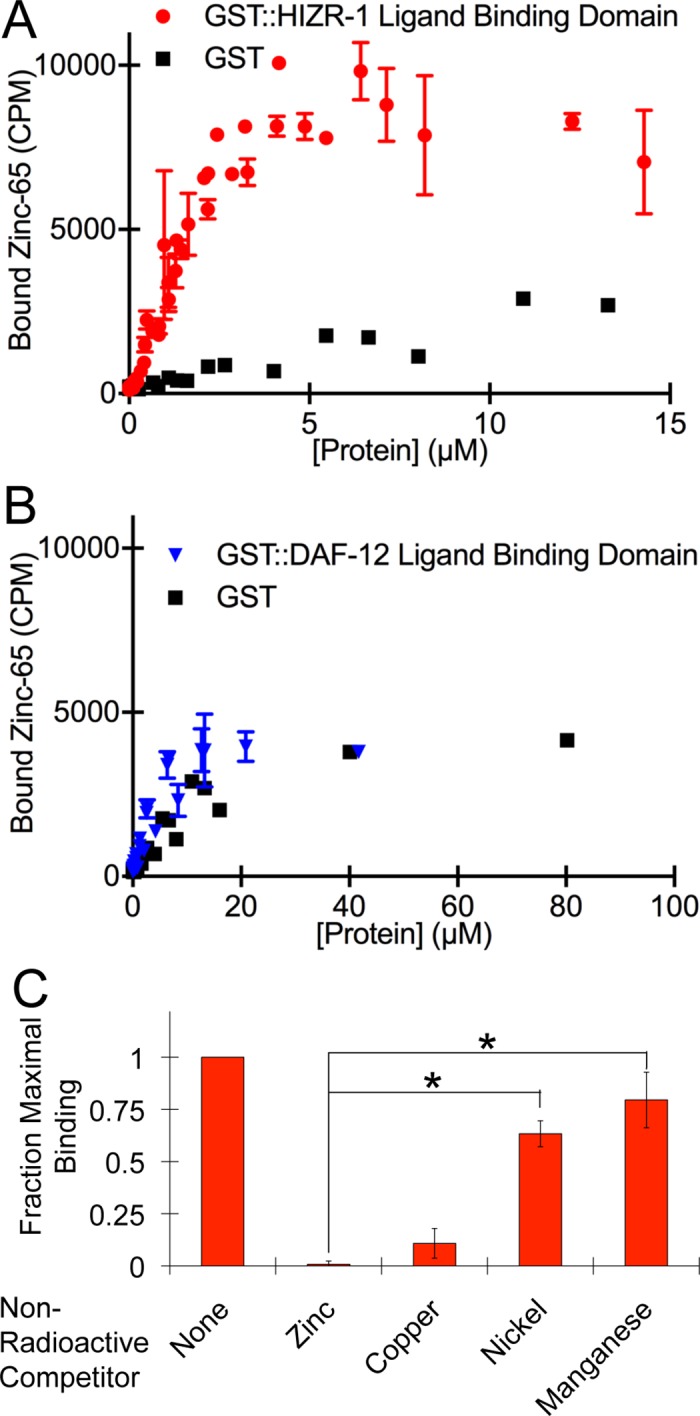
Zinc directly bound the HIZR-1 ligand-binding domain. **(A)** Glutathione S-transferase (GST) alone and the ligand-binding domain of HIZR-1 (residues 101–412) fused to GST were expressed in bacteria and partially purified by affinity chromatography. Increasing concentrations of either GST::HIZR-1(101–412 WT) or GST alone were incubated with a fixed concentration of radioactive zinc-65, and the amount of zinc-65 bound to protein was quantified by filter binding and scintillation counting. Values are the average +/- S.D. in counts per minute (CPM). At least two technical replicates were performed for each unique protein concentration. GST::HIZR-1(101–412 WT) displayed saturable binding, and a nonlinear regression was used to calculate a dissociation constant of 1.7 +/- 0.3 μM. X-ray crystallography studies indicate that GST binds one zinc molecule per protein [32]; our data are consistent with saturable, low-level zinc binding by GST alone. **(B)** GST alone and the ligand-binding domain of DAF-12 isoform A (residues 440–753) fused to GST were expressed in bacteria, partially purified by affinity chromatography, and analyzed for zinc binding using the method described above. GST again displayed saturable, low-level zinc binding. GST::DAF-12(440–753 WT) LBD displayed binding similar to GST alone, indicating that the LBD of DAF-12 does not bind an appreciable amount of zinc. **(C)** Partially purified GST::HIZR-1(101–412 WT) was incubated with radioactive zinc-65 and no additional metal (none) or 500 μM nonradioactive zinc, copper, nickel, or manganese. Bars indicate the amount of zinc-65 bound to protein +/- S.D. (*n* = 3) quantified by filter binding and scintillation counting. The values were normalized by setting the value of the sample with no additional metal equal to 1.0, defined as maximal binding. Low values indicate the nonradioactive metal competes effectively with radioactive zinc-65. For GST::HIZR-1(101–412 WT), compared to nonradioactive zinc, nickel and manganese displayed significantly lower effectiveness as competitors (*, *p* < 0.05). The value for copper was not significantly different than the value for zinc (*p* = 0.13), indicating copper effectively competes with zinc for binding.

We hypothesized that zinc is a ligand for HIZR-1, leading to the predictions that zinc will directly bind the LBD of purified HIZR-1 and high zinc will promote nuclear accumulation of HIZR-1 in animals. To avoid the complication of zinc binding to the DBD, which contains two predicted zinc finger motifs, we used affinity chromatography to partially purify the LBD of HIZR-1 fused to glutathione-S-transferase (GST). GST alone and GST fused to the LBD of the *C*. *elegans* DAF-12 NR, which uses dafachronic acid as a ligand [30,31], were used as specificity controls. The amino acid sequences of the DAF-12 LBD and the HIZR-1 LBD are about 15% identical, and about 47% of the residues are weakly similar. Zinc binding was analyzed using radioactive zinc-65. The LBD of HIZR-1 displayed saturable, high-affinity binding to zinc (**Fig 3A**). GST alone and the LBD of DAF-12 fused to GST displayed similar low-level binding, demonstrating the protein specificity of the zinc binding (**Fig 3B**). Nickel and manganese did not effectively compete with zinc for protein binding, demonstrating the interaction between the LBD of HIZR-1 and zinc was metal-selective (**Fig 3C**). However, copper did display binding to the LBD of HIZR-1. These results demonstrate a direct, high-affinity, protein-specific and metal-selective interaction between the LBD of HIZR-1 and zinc.

**Fig 4:**
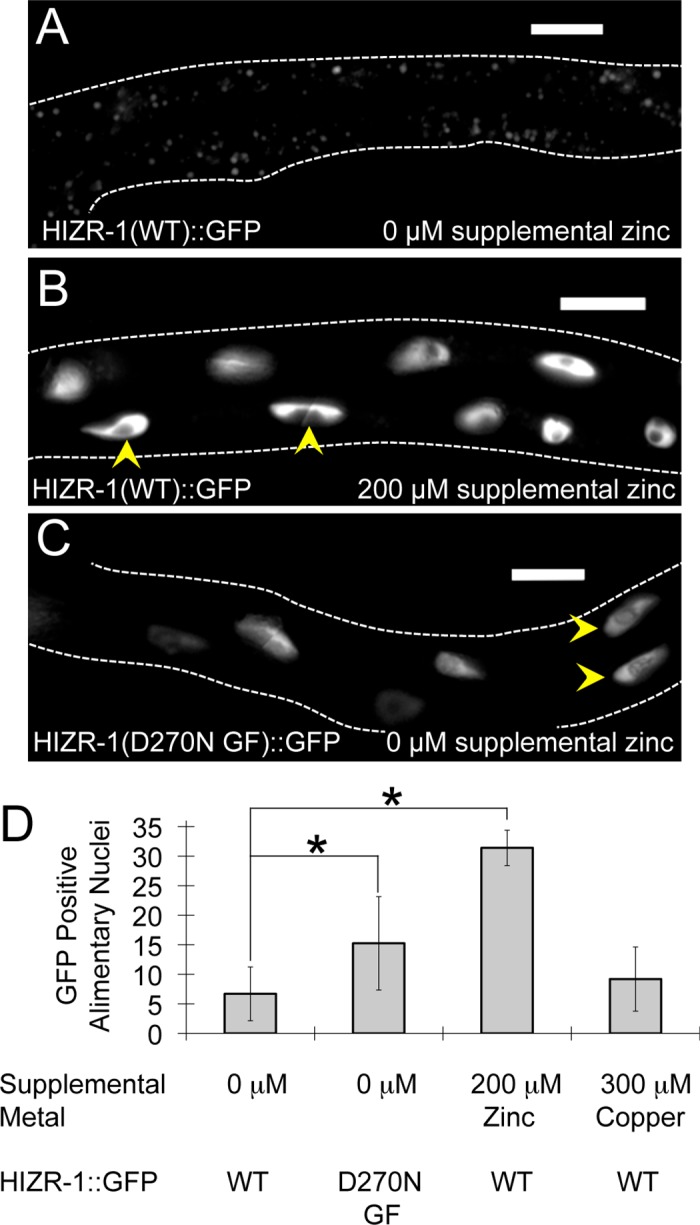
Zinc promoted nuclear accumulation of HIZR-1 in animals. *hizr-1(am286lf)* animals expressing full-length HIZR-1(1–412 WT)::GFP or HIZR-1(1–412 D270N GF)::GFP were cultured with no supplemental metals or supplemental zinc or copper. **(A–C)** Representative images show midbody region; the intestines are outlined, arrowheads indicate representative GFP-positive nuclei, and scale bars are approximately 10 μm. The correspondence of GFP fluorescence and intestinal cell nuclei was established by alternating between using differential interference contrast microscopy to visualize and identify nuclei based on morphology and location and fluorescence microscopy to visualize GFP. The punctate fluorescence in panel A appears to be autofluorescence derived from the gut granules, lysosome-related organelles that accumulate fluorescent material, and is not likely to result from HIZR-1(1–412 WT)::GFP expression. **(D)** Values are number of GFP-positive alimentary nuclei per animal +/- S.D. (*n* = 10–20) (*, *p* < 0.05).

These data can be used to estimate the stoichiometry of binding; however, this estimation is subject to important caveats. The crystal structure of GST indicates the protein binds one molecule of zinc, suggesting the stoichiometry of binding is 1 zinc:1 GST protein molecule [32]. If we assume this stoichiometry in our binding reactions, which has not been demonstrated directly, then we estimate the zinc:HIZR-1 LBD stoichiometry is 3:1 (**Fig 3A**) and 4:1 (**S3 Fig**). The amino acids that typically coordinate zinc in proteins are histidine and cysteine, and zinc is typically coordinated by four such residues. The predicted LBD of HIZR-1 contains 15 histidine and cysteine residues, suggesting it might have the capacity to coordinate three or four zinc ions with these residues. However, the data presented here do not address the role of these residues in zinc binding.

These data can be used to estimate a dissociation constant, although this value is subject to important caveats. The calculated dissociation constant was 2.6 +/- 0.2 μM when the concentration of zinc was varied (**S3 Fig**) and 1.7 +/- 0.3 μM when the concentration of protein was varied **(Fig 3A)**, which are in good agreement. However, several caveats must be considered in interpreting these calculated dissociation constant values: (1) The calculation assumes that all added zinc is available for protein binding; however, the binding reaction contained glutathionine, which is known to have zinc binding affinity and is predicted to reduce the concentration of zinc available to bind the protein. (2) The calculation assumes that every protein molecule is “active” and able to bind zinc, whereas some might have been inactive or denatured. (3) The calculation assumes that zinc and protein are bound in a 1:1 stoichiometry—as described above, HIZR-1 appears to bind more than one zinc per protein molecule. These data indicate that zinc directly binds the LBD of HIZR-1 with high affinity and metal selectivity. However, further biochemical studies are necessary to accurately define the dissociation constant, stoichiometry, and specific amino acid residues that mediate zinc binding.

### Zinc Promoted Nuclear Accumulation of HIZR-1 in the *C*. *elegans* Intestine

To characterize regulation in animals, we analyzed the HIZR-1(WT)::GFP protein that rescues the Zat-d phenotype, indicating it is functional and expressed in a pattern similar to endogenous protein. In animals cultured with no supplemental zinc, HIZR-1(WT)::GFP displayed low-level expression in intestinal cells; it was primarily localized in the cytoplasm and occasionally in nuclei. By contrast, culture in high dietary zinc resulted in striking HIZR-1(WT)::GFP accumulation in most nuclei of alimentary tract cells, primarily in the intestine (**Fig 4A,4B and 4D**) [33]. High dietary copper did not significantly affect the localization, demonstrating metal specificity of this response (**Fig 4D**).

To analyze the *am285* gain-of-function mutation, we generated animals that express mutant HIZR-1(D270N GF)::GFP protein. When cultured with no supplemental zinc, HIZR-1(D270N GF)::GFP animals displayed significantly more nuclear accumulation of GFP than HIZR-1(WT)::GFP animals (**Fig 4A,4C and 4D**). Thus, the D270N amino acid substitution is sufficient to promote nuclear accumulation of HIZR-1 and transcriptional activation of zinc-activated genes, highlighting the critical regulatory role of the LBD. Together, these results support the model that zinc binding to the LBD causes nuclear accumulation and transcriptional activation of HIZR-1.

### The HIZR-1 DNA-Binding Domain Directly Bound the HZA DNA Enhancer

**Fig 5:**
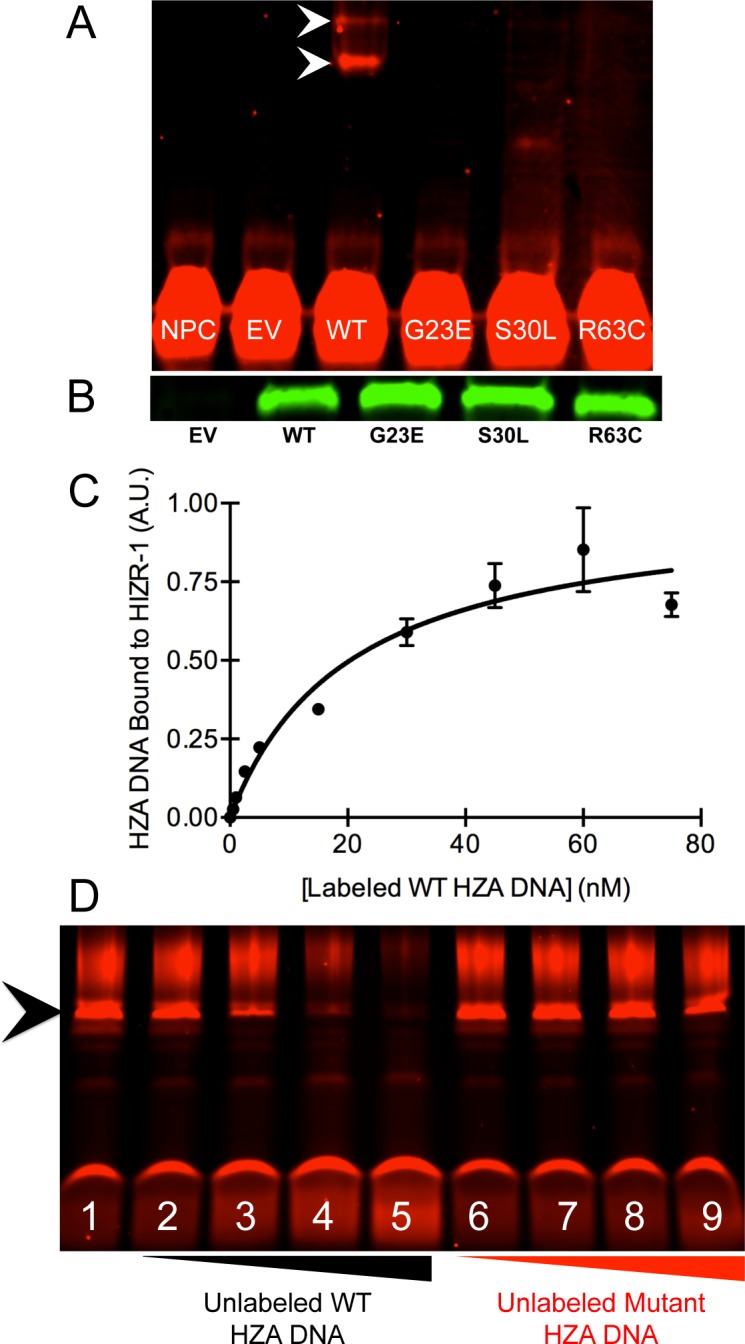
The HZA enhancer directly bound the HIZR-1 DNA-binding domain. **(A)** Infrared image of an electrophoretic mobility shift assay (EMSA). Labeled HZA enhancer DNA bound partially purified, full-length, wild-type (WT) HIZR-1 protein (arrowheads) but not mutant (G23E, S30L, or R63C) proteins. NPC, no protein control: EV, empty vector. The S30L lane contains a faint, rapidly migrating band of unknown significance. **(B)** Equivalent WT and mutant HIZR-1 protein amounts demonstrated by western blotting. **(C)** A constant amount of partially purified, full-length, wild-type HIZR-1 protein was incubated with variable concentrations of labeled, wild-type HZA DNA. Values are the average amount of labeled HZA DNA that displayed retarded migration +/- S.D. determined by image analysis and expressed in arbitrary units (A.U.). At least two technical replicates were performed for each HZA DNA concentration. Retarded migration indicates the DNA is bound to HIZR-1 protein. Nonlinear regression was used to calculate a dissociation constant of 20.4 +/- 6.8 nM. **(D)** A constant amount of partially purified, full-length, wild-type HIZR-1 protein was incubated with a constant amount of labeled wild-type HZA enhancer DNA. The interaction between the labeled DNA and HIZR-1 protein was competed with no unlabeled DNA (Lane 1) or increasing concentrations (5, 50, 500, or 5,000 nM) of unlabeled wild-type HZA DNA (Lanes 2–5) and unlabeled mutant HZA DNA (Lanes 6–9). The mutant HZA DNA had the identical flanking sequences but the order of the 15 base pair HZA was randomized. Lanes 3–5 display competition of binding, as evidenced by diminishment of the intensity of the retarded band (arrowhead). By contrast, lanes 7–9 do not display diminishment of the intensity of the retarded band, indicating that the mutant HZA DNA does not compete for binding to the HIZR-1 protein.

We hypothesized that HIZR-1 directly binds the HZA enhancer to mediate transcriptional activation, leading to the predictions that HZA enhancer DNA will interact directly with the DBD of purified HIZR-1 and that the HZA mediates the transcriptional activation activity of HIZR-1 in animals. An electrophoretic mobility shift assay (EMSA) was conducted using partially purified, full-length HIZR-1 protein and fluorescently labeled DNA. The 35 base pair DNA sequence was derived from the *cdf-2* promoter and included the 15 base pair HZA with 10 flanking base pairs on each side. HIZR-1 protein retarded the migration of the HZA DNA in the gel, indicating a direct interaction (**Fig 5A and 5B**). The binding was saturable, and an apparent dissociation constant of 20.4 +/- 6.8 nM was calculated (**Fig 5C**). Unlabeled wild-type (WT) and mutant HZA were used as specificity controls. Unlabeled WT HZA was identical in sequence to the fluorescently labeled HZA while the unlabeled mutant HZA was identical except for randomizing the order of the central 15 base pair HZA. Unlabeled mutant HZA DNA did not effectively compete for binding to HIZR-1, indicating the binding activity is sequence specific (**Fig 5D**). These data demonstrate a direct, high-affinity, sequence-specific interaction between HIZR-1 and the HZA enhancer.

To investigate *hizr-1(lf)* mutations that result in substitutions of highly conserved amino acids in the DBD, we conducted EMSA assays with mutant proteins. All three mutant proteins displayed dramatically reduced DNA binding (**Fig 5A and 5B**). These biochemical and genetic analyses indicate that the DBD of HIZR-1 mediates the interaction with HZA DNA and DNA binding is necessary for the transcriptional response to high dietary zinc in animals.

### HIZR-1 Acted through the HZA DNA Enhancer In Vivo

**Fig 6:**
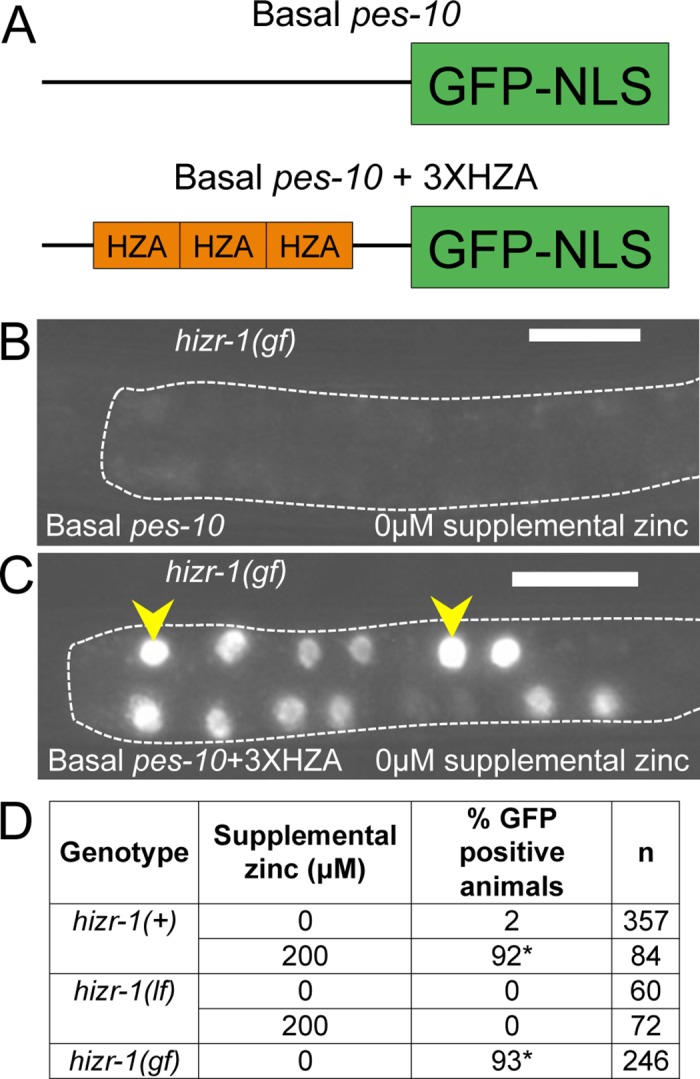
The HZA enhancer mediated the transcriptional activity of HIZR-1 in animals. **(A)** Diagrams of the basal *pes-10* promoter with and without three HZA enhancer elements (not to scale) [15]. **(B,C)** Representative images of anterior intestine of *hizr-1(am285gf)* transgenic animals containing *pes-10p*::*gfp-nls* (basal *pes-10*) or *3XHZApes-10p*::*gfp-nls* (basal *pes-10*+3X HZA); intestines are outlined, arrowheads indicate representative GFP-positive nuclei, and scale bars are approximately 25 μm. **(D)** *hizr-1(+)*, *hizr-1(am286lf)*, and *hizr-1(am285gf*) transgenic animals expressing *3XHZApes-10p*::*gfp-nls* were cultured with 0 or 200 μM supplemental zinc and assayed for GFP expression. n, number of animals assayed, *, *p* < 0.05 compared to *hizr-1(+)* animals cultured with 0 μM supplemental zinc (Chi-squared test).

To investigate the role of the HZA enhancer in animals, we utilized a promoter construct that contains three copies of the HZA enhancer upstream of a basal *pes-10* promoter driving expression of GFP with a nuclear localization sequence (**Fig 6A**) [15]. Computational analysis revealed that the basal *pes-10* promoter does not contain a recognizable HZA element, and experimental analysis demonstrated that the basal *pes-10* promoter is not activated by high zinc [15]. In *hizr-1(+)* transgenic animals, 2% displayed GFP when cultured with no supplemental zinc, whereas 92% displayed GFP in high dietary zinc, demonstrating the promoter is significantly activated by high zinc. By contrast, 0% of *hizr-1(am286lf)* mutant animals displayed GFP in high dietary zinc, significantly less than *hizr-1(+)* (*p* < 0.001 by Chi-squared test). Furthermore, 93% of *hizr-1(am285gf)* mutant animals displayed GFP induction when cultured with no supplemental zinc, significantly more than *hizr-1(+)* (**Fig 6B–6D, S4 Fig**). These data show that *hizr-1* was necessary and sufficient for high-zinc–activated transcription mediated by the HZA enhancer in animals.

### HIZR-1 Activated Transcription of Its Own mRNA in Response to High Zinc, Defining a Positive Feedback Circuit

**Fig 7:**
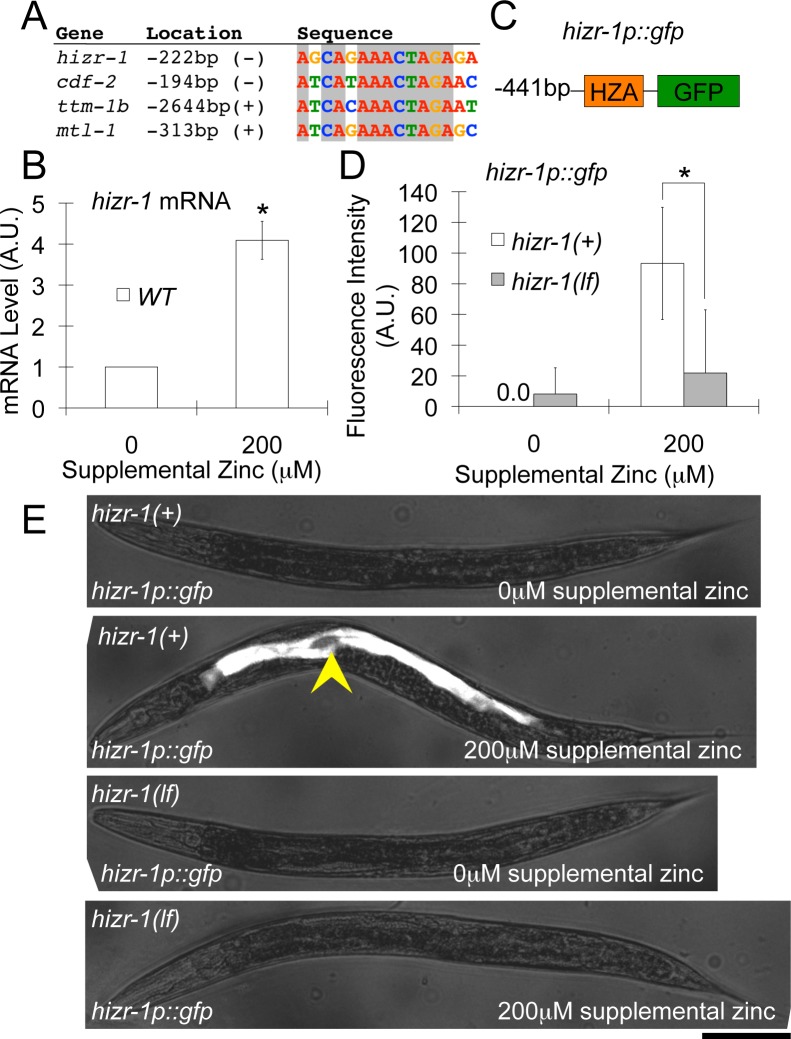
The *hizr-1* promoter contains a HZA enhancer and is activated by high dietary zinc, which requires *hizr-1* function. **(A)** An alignment of the 15 bp DNA sequence of HZA elements in *hizr-1*, *cdf-2*, *ttm-1b*, and *mtl-1;* 11 positions are identical in these four sequences (boxed). The location is the first nucleotide of the DNA motif; the A residue of the translation start site (ATG) is defined as +1; (+) and (-) indicate the same and opposite orientation relative to the direction of transcription, respectively. **(B)** mRNA was isolated from populations of wild-type (white) animals cultured with 0 or 200 μM supplemental zinc. *hizr-1* transcript levels were analyzed by qPCR; mRNA levels are expressed in arbitrary units and were normalized to *rps-23*, a ribosomal protein gene that is not regulated by high zinc. The values were normalized by setting the value for wild-type animals at 0 μM supplemental zinc equal to 1.0. Bars represent the average +/- S.D. (*n* = 3), (*, *p* < 0.05). *hizr-1* transcript levels were increased significantly when cultured with 200 μM supplemental zinc compared to 0 μM supplemental zinc, demonstrating that it is a high-zinc–activated transcript. **(C)** A diagram (not to scale) of the *hizr-1p*::*gfp* transcriptional reporter construct containing the *hizr-1* promoter (black line, 441 bp upstream of the ATG start codon) fused to the coding region of green fluorescent protein (green box). The *hizr-1* promoter contains a HZA enhancer element (orange box). **(D, E)** Transgenic *hizr-1(+)* (white) or *hizr-1(am286lf)* (gray) animals expressing the *hizr-1p*::*gfp* transcriptional reporter were cultured with 0 or 200 μM supplemental zinc. **(D)** GFP fluorescence in the intestine was quantified by image analysis. Bars represent the average fluorescence intensity +/- S.D expressed in arbitrary units (A.U.) (*n* = 8–10 animals), (*, *p* < 0.05). In *hizr-1(+)* animals, GFP expression levels were increased significantly when cultured with 200 μM supplemental zinc compared to 0 μM supplemental zinc, demonstrating that the *hizr-1* promoter is activated by high zinc. Compared to *hizr-1(+)* animals, *hizr-1(am286lf)* mutant animals displayed significantly lower GFP expression when cultured with 200 μM supplemental zinc, indicating a defect of high-zinc–activated transcription. **(E)** Images of representative transgenic animals. Intestinal fluorescence is indicated by the arrowhead and required both high dietary zinc and *hizr-1* function. Scale bar is approximately 100 μm.

**Fig 8:**
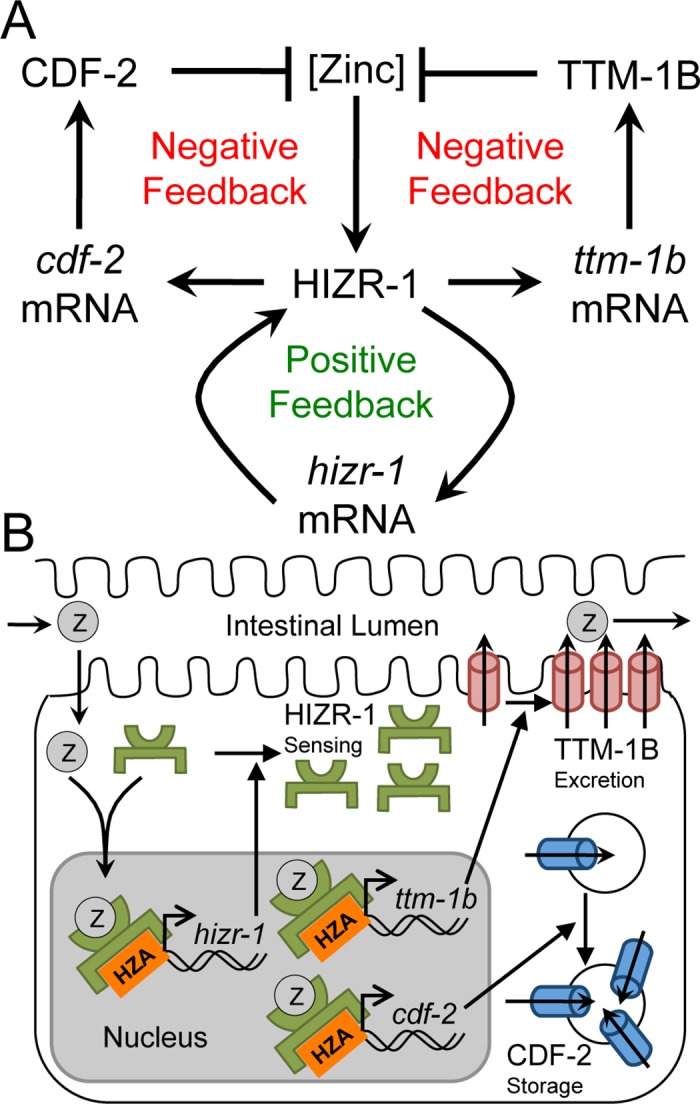
HIZR-1 regulates high zinc homeostasis in the *C*. *elegans* intestine. **(A)** Genetic model. High levels of zinc promote HIZR-1 activity and transcriptional activation of multiple genes including *cdf-2*, *ttm-1b* and *hizr-1*. Increased levels of *cdf-2* and *ttm-1b* mRNA promote increased levels of CDF-2 and TTM-1B protein, which reduce levels of cytoplasmic zinc in a parallel negative feedback circuit. Increased levels of *hizr-1* mRNA promotes increased levels of HIZR-1 protein, creating a positive feedback circuit that enhances the negative feedback system. **(B)** Molecular model. Dietary zinc (Z) enters intestinal cells, binds the LBD of HIZR-1 and promotes nuclear accumulation, HZA enhancer binding, and transcriptional activation. The nuclear accumulation of HIZR-1 could result from increased HIZR-1 protein levels due to autoregulation and/or translocation of HIZR-1 from the cytoplasm to the nucleus. Increased levels of CDF-2 and TTM-1B promote zinc detoxification by sequestration in lysosome-related organelles [13] and excretion into the intestinal lumen [14], respectively. Increased levels of HIZR-1 promote homeostasis by a positive feedback circuit.

In *C*. *elegans*, high zinc homeostasis is mediated by a parallel negative feedback circuit; high levels of cytoplasmic zinc increase expression of CDF-2 and TTM-1B, which detoxify zinc by sequestration and excretion, respectively [13,14]. Here we show that HIZR-1 plays a pivotal role in this negative feedback circuit by sensing zinc levels, binding the HZA enhancer, and promoting transcriptional activation of these genes. We noticed that the promoter of *hizr-1* contains a predicted HZA element (**Fig 7A**), leading to the hypothesis that HIZR-1 activates transcription of its own promoter. Consistent with this model, the level of *hizr-1* mRNA was significantly increased about 4-fold by high dietary zinc in wild-type animals (**Fig 7B**). This regulation appears to occur at the level of transcription, since a construct containing the *hizr-1* promoter driving expression of GFP (*hizr-1p*::*gfp*) was also induced by high dietary zinc (**Fig 7C–7E**). By contrast, *hizr-1(am286lf)* transgenic animals containing this construct did not display increased GFP expression in response to high dietary zinc, indicating that *hizr-1* is necessary for this transcriptional activation (**Fig 7D and 7E**). These results identify a positive feedback circuit, since HIZR-1 protein increases levels of *hizr-1* mRNA, which in turn increases levels of HIZR-1 protein. This positive feedback circuit is embedded in and promotes the parallel negative feedback circuits—increased levels of HIZR-1 protein will enhance the activation of *cdf-2* and *ttm-1b* mRNA, promoting zinc homeostasis (**Fig 8**).

## Discussion

### A New High Zinc Sensor in Animals

Sensing high and low levels of zinc is critical for homeostasis. Here we identify the nuclear receptor HIZR-1 as a high zinc sensor in an animal. The DNA-binding domain of HIZR-1 interacted directly with the HZA enhancer in purified extracts, and *hizr-1* functioned through the HZA enhancer in vivo to mediate transcriptional activation in response to high zinc. Thus, HIZR-1 appears to be the HZA-binding factor that was postulated by Roh et al. (2015) when the HZA was identified as the enhancer that mediates transcriptional activation in response to high dietary zinc [15]. Nuclear receptors are typically regulated by ligand binding, which promotes nuclear accumulation and transcriptional activation [27]. We propose that a ligand for HIZR-1 is zinc, and this model is supported by two lines of evidence. First, HIZR-1 accumulated in the nucleus and activated transcription in response to high dietary zinc. Second, the ligand-binding domain of HIZR-1 interacted directly with zinc with high affinity in purified extracts. This zinc affinity of the ligand-binding domain is specific for HIZR-1, since the related ligand-binding domain of DAF-12, which uses dafachronic acid as a ligand, did not bind zinc. Furthermore, our genetic studies demonstrate that the ligand-binding domain of HIZR-1 plays a key regulatory role, since a missense mutation in the domain causes a gain-of-function phenotype characterized by constitutive nuclear accumulation and transcriptional activation. The DNA-binding domain of HIZR-1 contains two predicted zinc finger motifs that are likely to bind zinc and promote DNA binding. While the results indicate the ligand-binding domain plays a critical regulatory role, the data do not exclude the possibility that zinc interactions with the DNA-binding domain also regulate HIZR-1 activity. These results establish the function and mechanism of action of *hizr-1* as the direct sensor of high zinc and the effector of high-zinc–activated transcription.

Our results raise the possibility that HIZR-1 might respond to multiple metal ions. Metal binding by the HIZR-1 LBD binding was relatively metal-specific, as nickel and manganese did not compete effectively with zinc for binding. Interestingly, copper was able to compete for binding to the HIZR-1 LBD, similar to zinc. However, multiple lines of evidence suggest that copper is not a functional ligand for HIZR-1: (i) *hizr-1(lf)* animals were not hypersensitive to high copper toxicity, (ii) high copper did not stimulate nuclear accumulation of HIZR-1, and (iii) high copper did not induce the transcription of *cdf-2*, *ttm-1b*, or *mtl-1* [15]. Cadmium is similar to zinc, but it is an environmental pollutant rather than a physiological metal. Cadmium activates gene transcription in worms, including some genes that contain HZA elements and also respond to zinc. Further studies are necessary to determine the role of HIZR-1 in cadmium-activated transcription.

Transcription factors that play a role in zinc homeostasis have been characterized in several eukaryotic organisms. In the budding yeast *Saccharomyces cerevisiae* the response to low zinc is mediated by the zinc-responsive activator protein 1 (ZAP1). The ZAP1 transcription factor binds directly to a conserved DNA element in promoter regions called the zinc-responsive element (ZRE) and thereby induces target gene expression [34]. ZAP1 target genes include the zinc importers ZRT1 and ZRT2 that are induced by ZAP1 to promote zinc uptake; ZRT3 is induced to mobilize zinc stored in the vacuole [35]. ZAP1 activity is repressed by high zinc [24] and activated by low zinc; this transcription factor contains multiple zinc finger domains that may play a regulatory role. In the fission yeast *Schizosaccharomyces pombe*, gene repression in zinc-replete cells is mediated by the Loz1 transcription factor [36,37]. The response to high zinc has been characterized in animals based on studies of the metallothionein genes. In mammals, a zinc-finger containing transcription factor called the metal-responsive-element-binding transcription factor-1 (MTF-1) directly binds the metal response element (MRE) in the promoters of metallothionein genes [16,25,38,39]. MTF-1 is necessary for the transcriptional response to a wide range of stresses including high cadmium, high zinc, hypoxia and oxidative stress caused by reactive oxygen species. The mechanisms of MTF-1 regulation are controversial, and it is unclear whether MTF-1 senses zinc directly or is part of a system that includes another high zinc sensor [40,41]. HIZR-1 is a new type of zinc sensor, and its discovery and characterization represent important advances in understanding mechanisms of high zinc homeostasis.

### A Homeostatic System for High Zinc: A Positive Feedback Loop Embedded in a Parallel Negative Feedback Circuit

In response to fluctuating zinc levels, organisms maintain zinc homeostasis by regulating the abundance and activity of metallothioneins and zinc transporters. In *C*. *elegans*, exposure to high levels of zinc causes induction of CDF-2, which sequesters zinc in lysosome-related organelles, and TTM-1B, which excretes zinc into the intestinal lumen. CDF-2 and TTM-1B function in a parallel negative feedback circuit, since single mutants display moderate or undetectable hypersensitivity to high zinc, respectively, whereas double mutants display dramatic hypersensitivity to high zinc [14]. Here we elucidate new aspects of the homeostatic system by showing that *hizr-1* mediates the transcriptional response of *cdf-2* and *ttm-1b*. Furthermore, HIZR-1 activates transcription of its own mRNA, thereby establishing a positive feedback loop: high dietary zinc increases the activity of HIZR-1 protein, which increases the levels of *hizr-1* mRNA and protein, resulting in a further increase in HIZR-1 activity. Because this positive feedback loop is embedded in a negative feedback circuit, it serves to enhance the overall negative feedback circuit.

The transcription factor ZAP1 is a key part of a similar feedback circuit in response to low zinc in yeast. Zinc deficiency causes activation of ZAP1 protein, which binds the promoter and activates transcription of the Zap1 gene; this autoregulation is a positive feedback loop [42]. Activated ZAP1 protein also increases the transcription of key zinc importers such as ZRT1 and ZRT2, representing the negative feedback component of this system[43,44]. The similarities between the circuits controlled by HIZR-1 in response to high zinc in animals and ZAP1 in response to low zinc in yeast highlights the utility and importance of embedding a positive feedback loop within negative feedback circuits to maintain homeostasis.

### Zinc Represents a New Class of Ligand for Nuclear Receptors

The discovery of HIZR-1 establishes a new intersection between two important fields that were previously separate: nuclear receptors and zinc biology. Nuclear receptors were originally defined as endocrine sensors in humans, including the glucocorticoid receptor and estrogen receptor [45,46]. Genome analysis identified a nuclear receptor superfamily consisting of about 49 members in mammals. Despite decades of intensive efforts focused on ligand discovery, about half of the nuclear receptors remain “orphans” with unknown physiological ligands. This raises the possibility that novel classes of ligands remain to be discovered. Indeed, the demonstration that zinc functions as a ligand for the HIZR-1 nuclear receptor represents the first example of a new class of physiological metal ligands. All previously described physiological ligands for nuclear receptors are small hydrophobic molecules; established ligand classes include retinoids [47,48], steroids [49], sterols [30,50], fatty acid derivatives [51], and other organic molecules, such as heme [52]. Interestingly, several metals have also been reported to bind to the LBD of the estrogen receptor and appear to alter its activity; however, these metalloestrogens are typically classified as endocrine disruptors rather than physiological ligands for the estrogen receptor [53]. The demonstration that a transition metal ion is the physiological ligand establishes a new structural class of nuclear receptor ligand molecules. Furthermore, this finding raises the possibility that nuclear receptors may be sensors of high levels of other essential metal ions, such as iron, copper, and manganese.

### Direct Nutrient Sensing Is a New Physiological Function for Nuclear Receptors

Nuclear receptors are not present in single-celled eukaryotes, such as yeast, and appear to have evolved in primitive multicellular organisms. Ancestral nuclear receptor genes exist in sponges, animals of the phylum Porifera, which lack higher-level body organization such as tissues and organs and thus lack hormone signaling [28,54]. Therefore, nuclear receptors were proposed to have ancestral roles as environmental sensing proteins that later evolved to sense intraorganismal endocrine signals [28]. However, no such functions have been rigorously demonstrated. Our results document a nuclear receptor that responds to a nutrient, consistent with the theory that the ancestral function of nuclear receptors might have been sensing dietary and environmental stimuli, including metals such as zinc. This represents a distinct paradigm from canonical endocrinology in which a hormone like estrogen is synthesized in endocrine cells, is secreted into the bloodstream, and enters distant cells, where it binds and activates the estrogen receptor [55]. Furthermore, this is a novel demonstration that a dietary nutrient is a direct ligand for a nuclear receptor. This establishes a new paradigm for nuclear receptors as direct sensors of environmental nutrients.

## Materials and Methods

### General Methods and Strains

*C*. *elegans* strains were cultured at 20°C on nematode growth medium (NGM) seeded with *Escherichia coli* OP50 unless otherwise noted [56]. The wild-type strain was Bristol N2. The Zat mutations *hizr-1(am279)*, *hizr-1(am280)*, *hizr-1(am285)*, *hizr-1(am286)*, *hizr-1(am287)*, and *hizr-1(am288)* are described here. These mutations were generated by mutagenizing the high-zinc reporter strain WU1391 (*cdf-2p*::*gfp*, see Plasmid DNA construction and transgenic strain generation for details) with ethyl methanesulfonate (EMS) [56] and identified by screening for abnormal patterns of fluorescence, as described below. *hizr-1(gk698405)* was identified by the *C*. *elegans* million mutations project and obtained from the *Caenorhabditis* Genetics Center [29]. To position newly identified mutations in the *C*. *elegans* genome, we used the following mutations on linkage group X that cause visible phenotypes: *unc-115(e2225)*, *egl-15(n484)* [57], and *sma-5(n678)*. To generate the high-zinc reporter strain WU1391, we utilized the *unc-119(ed3)* mutation [58].

### Plasmid DNA Construction and Transgenic Strain Generation

To generate the transcriptional fusion constructs for *cdf-2* (*cdf-2p*::*gfp*) (pSC24) and *hizr-1* (*hizr-1p*::*gfp*) (pKW11), we polymerase chain reaction (PCR)-amplified DNA fragments positioned upstream of the coding sequence using wild-type *C*. *elegans* DNA. These fragments were ligated into pBluescript SK+ (Stratagene) containing the green fluorescent (GFP) coding sequence and the *unc-54* 3' untranslated region (UTR). The *cdf-2* promoter was amplified from the ATG start codon to 1,371 base pairs upstream of the ATG start codon. The *hizr-1* promoter was amplified from the ATG start codon to 441 base pairs upstream of the ATG start codon. To analyze the HZA enhancer, we used previously described transcriptional reporter constructs containing the basal *pes-10* promoter driving transcription of GFP with a nuclear localization sequence (NLS) (*pes-10p*::*gfp-nls*) (pPD107.94, a gift from A. Fire) and the basal *pes-10* promoter with three copies of the HZA enhancer inserted into the promoter (*3XHZApes-10p*::*gfp-nls*) (pID24) [15].

To generate the translational fusion construct for HIZR-1, [HIZR-1(1–412 WT)::GFP] (pKW1), we PCR-amplified the *hizr-1* genomic locus from the *C*. *elegans* fosmid WRM069cE11. This fragment was ligated into pBluescript SK+ (Stratagene) containing the GFP coding sequence and the *unc-54* 3' UTR. The *hizr-1* locus was amplified from 444 base pairs upstream of the ATG start codon to the TAA stop codon. The TAA stop codon was mutated to TAT to allow translation of the C-terminal GFP. To generate the HIZR-1(1–412 D270N GF)::GFP construct (pKW8), we modified plasmid pKW1 using Agilent QuickChange II Site-Directed Mutagenesis Kit according to manufacturer’s instructions.

To integrate the *cdf-2p*::*gfp* transcriptional fusion construct into the *C*. *elegans* genome, we ligated the DNA fragment encoding the *cdf-2* promoter driving expression of GFP with the *unc-54* 3' UTR into the plasmid pMM016 that contains the wild-type *unc-119* locus (*unc-119(+)*) [59] (pSC24). pSC24 was bombarded into *unc-119(ed3)* animals [13,59], and nonUnc animals that segregated only nonUnc self progeny were selected. The *cdf-2p*::*gfp unc-119(+)* transgene is integrated on the right arm of linkage group IV and was assigned the allele name *amIs10*. The following transgenic strains with *amIs10* were used in this study: WU1391 (*cdf-2p*::*gfp* outcrossed seven times to N2), WU1518 (*cdf-2p*::*gfp* outcrossed seven times to Hawaiian CB4856), *cdf-2p*::*gfp; hizr-1(am279)*, *cdf-2p*::*gfp; hizr-1(am280)*, *cdf-2p*::*gfp; hizr-1(am285)*. *cdf-2p*::*gfp;hizr-1(am286)*, *cdf-2p*::*gfp; hizr-1(am287)*, and *cdf-2p*::*gfp; hizr-1(am288)*.

Transgenic animals containing extrachromosomal arrays were generated by injecting the gonad of worms with a plasmid of interest (pKW1, pKW8, pKW11, pID24, or pPD107.94) and a co-injection marker [60]. The co-injection marker was either *myo-3p*::*mCherry* (pCJF104) [61] or the plasmid pRF4 encoding the dominant ROL-6(R71C GF) mutant protein [60]. Transgenic animals were selected by mCherry expression in body-wall muscles or by the Rol phenotype. The following transgenic strains with extrachromosomal arrays were used in this study: *hizr-1(am286);* HIZR-1(1–412 WT)::GFP, *hizr-1(am286);* HIZR-1(1–412 D270N GF)::GFP, *hizr-1(+); 3XHZApes-10p*::*gfp-nls*, *hizr-1(am286); 3XHZApes-10p*::*gfp-nls*, *hizr-1(am285); 3XHZApes-10p*::*gfp-nls*, *hizr-1(am285); pes-10p*::*gfp-nls*, *hizr-1(+); hizr-1p*::*gfp*, and *hizr-1(am286); hizr-1p*::*gfp*. All transgenic strains contained the pRF4 dominant Rol marker except for *hizr-1(+); 3XHZApes-10p*::*gfp-nls* which contained the *myo-3p*::*mCherry* marker.

To generate plasmid constructs for protein purification of full-length HIZR-1, we PCR-amplified the complete coding sequence of HIZR-1 from synthesized DNA (IDT gBlocks). This fragment was ligated into pTrcHisA (ThermoFisher). This plasmid encodes an N-terminal 6 histidine affinity purification tag (6XHis) fused to amino acids 1–412 of HIZR-1 (pKW2). The pKW2 plasmid was modified using the Agilent QuickChange II Site-Directed Mutagenesis Kit according to manufacturer’s instructions to generate the plasmids pKW4, pKW5, and pKW6 that encode full-length HIZR-1 proteins with the G23E, S30L, and R63C amino acid substitutions, respectively.

To generate plasmid constructs for protein purification of the ligand-binding domains of HIZR-1 and DAF-12, we PCR-amplified the ligand-binding domains of HIZR-1 (encoding amino acids 101–412) and DAF-12 (encoding amino acids 440–753 of the A isoform) from synthesized DNA (IDT gBlocks). These fragments were ligated into pGEX-4T-1 (GE Healthcare). These plasmids encode an N-terminal glutathione S-transferase (GST) affinity purification tag fused to either the ligand-binding domain of HIZR-1 (pKW14) or DAF-12 (pKW15).

All plasmid constructs were verified by DNA sequencing using standard methods.

### Positioning Mutations in the Genome

To position Zat mutations on the genetic map, we utilized single nucleotide polymorphism (SNP) markers [18]. To facilitate this strategy, we introduced the integrated *cdf-2p*::*gfp* reporter from WU1391 into Hawaiian CB4856 by performing seven backcrosses to CB4856 with selection for zinc-activated GFP fluorescence. All six Zat-c and Zat-d mutations displayed tightest linkage to the SNP *amP117*, positioned at approximately the 9,552 kilobase pair on linkage group X (**Fig 1E**).

To determine how many genes were affected by the five recessive Zat-d mutations, we performed complementation experiments. All five mutations failed to complement one another for the Zat-d phenotype, indicating that all five mutations affect the same gene.

To define intervals that contain the Zat-c mutation (*am285)* and the Zat-d complementation group (represented by *am286)*, we conducted three-factor mapping experiments with mutations that cause visible phenotypes [56]. We chose the X-linked genes *unc-115*, *egl-15*, and *sma-5* that are located at approximately the 10,147, 11,016, and 12,005 kilobase pairs on linkage group X, respectively. The *cdf-2p*::*gfp* reporter was introduced into the double mutant mapping strains to generate *cdf-2p*::*gfp*; *unc-115(e2225) egl-15(n484)* or *cdf-2p*::*gfp*; *egl-15(n484) sma-5(678)* using standard genetic techniques. For the *am285* Zat-c mutation, 0/13 Egl nonUnc and 5/5 Unc nonEgl recombinants segregated the Zat-c phenotype, indicating *am285* is positioned right of *egl-15*. 9/20 Egl nonSma recombinants segregated the Zat-c phenotype, indicating *am285* is positioned between *egl-15* and *sma-5*. For the *am286* Zat-d mutation, 0/12 Egl nonUnc and 3/3 Unc nonEgl recombinants segregated the Zat-d phenotype, indicating *am286* is positioned right of *egl-15*. 9/30 Egl nonSma recombinants segregated the Zat-d phenotype, indicating *am286* is positioned between *egl-15* and *sma-5*. The results that 18/50 recombination events (36%) occurred between *egl-15* and *hizr-1* and 32/50 recombination events (64%) occurred between *hizr-1* and *sma-5* are consistent with the molecular distances of approximately 302 kilobase pairs between *egl-15* and *hizr-*1 (31%) and 687 kilobase pairs between *hizr-1* and *sma-5* (69%).

To identify the gene affected by the newly isolated Zat mutations, we performed whole genome sequencing using DNA from the *am279*, *am280*, *am285*, and *am286* mutant strains. Candidate mutations within the mapping interval were identified by comparing the mutant DNA sequence to wild-type DNA sequence. *am279*, *am280*, *am285*, and *am286* all contained candidate mutations in the gene *hizr-1/*(*ZK455*.*6*) (**Fig 1E, S1 Table**). Next, the sequence of the *hizr-1* genomic locus was determined by standard sequencing techniques using DNA from *am287* and *am288* mutant strains, which revealed mutations in the *hizr-1* locus.

### mRNA Analyses

RNA isolation and cDNA synthesis were performed as previously described [19]. Mixed-developmental stage populations of animals were cultured on NGM dishes. Animals were washed and cultured on Noble agar minimal media (NAMM) dishes with and without supplemental zinc sulfate. NAMM dishes were seeded with concentrated OP50 *E*. *coli*. After 16–24 h of culture on NAMM dishes, animals were collected by washing for RNA extraction. RNA was extracted using TRIzol (Invitrogen), treated with DNase I, and cDNA was synthesized using the High-Capacity cDNA Reverse Transcription kit (Applied Biosystems) according to the manufacturer’s instructions. Quantitative PCR (qPCR) was performed using a 7900HT Fast Real-Time PCR system (Applied Biosystems) and the SYBR Green PCR Master Mix (Applied Biosystems) following the manufacturer’s instructions. mRNA fold change was calculated using the comparative C_T_ method [62]. For all qPCR experiments, mRNA levels were normalized to *rps-23* and error bars indicate standard deviation. Forward and reverse amplification primers were: *rps-23* 5'-aaggctcacattggaactcg and 5'-aggctgcttagcttcgacac; *cdf-2* 5'-atagcaatcggagagcaacg and 5'-tgtgacaattgcgagtgagc; *ttm-1b* 5'-catgggcactcacacacacac and 5'-ctcggcgacccttttgatatttc; *hizr-1* 5'-tcattttgcggtttcatcgtg and 5'-catcgcgtgtatctacagctac; and *mtl-1* 5'-ggcttgcaagtgtgactgc and 5'-cctcacagcagtacttctcac.

### Growth Rate Assay

Synchronized embryos were placed on dishes containing NAMM that were seeded with concentrated OP50 *E*. *coli* and supplemented with zinc sulfate (ZnSO_4_) or copper chloride (CuCl_2_) and cultured for 3 d [18]. Animals were then mounted on 2% agarose pads on microscope slides and imaged with a Zeiss Axioplan 2 microscope equipped with a Zeiss AxioCam MRm digital camera. The length of individual animals (tip of head to end of tail) was measured using ImageJ software (NIH).

### HIZR-1 Expression Pattern and Fluorescence Microscopy

Live *hizr-1(am286);* HIZR-1(1–412 WT)::GFP or *hizr-1(am286);* HIZR-1(1–412 D270N GF)::GFP transgenic animals (L4 or young adult) were washed and cultured on NAMM dishes with or without zinc sulfate for 12–16 h. Transgenic animals were then immobilized and mounted onto a microscope slide with a thin pad of 2% agarose. All images were captured using a Zeiss Axioplan 2 microscope equipped with a Zeiss AxioCam MRm digital camera using identical settings and exposure times in paired experiments (**Fig 4A–4C**). To score alimentary nuclei per animal with detectable HIZR-1::GFP (**Fig 4D**), we examined live animals using an Olympus SZX12 dissecting microscope equipped with GFP fluorescence and counted GFP-positive alimentary nuclei. Animals were exposed to 200 μM zinc or 300 μM copper because they are approximately equally toxic to *C*. *elegans* [63].

Transgenic L4 or young adult animals expressing *cdf-2p*::*gfp* (**Fig 1A and 1B**) or *hizr-1p*::*gfp* (**Fig 7C–7E**) were cultured for 12–16 h on NAMM dishes with or without supplemental zinc sulfate. Transgenic animals were immobilized, mounted, and imaged as described above. GFP fluorescence intensity was quantified using ImageJ software (NIH).

To determine the percent of GFP-positive animals in a population of transgenic animals expressing *P3XHZApes-10p*::*gfp-nls* or *pes-10p*::*gfp-nls*, we examined live animals using an Olympus SZX12 dissecting microscope equipped with GFP fluorescence. Animals displaying one or more GFP-positive alimentary nuclei were classified as GFP positive (**Fig 6D**). Representative images were captured using a Zeiss Axioplan 2 microscope equipped with a Zeiss AxioCam MRm digital camera using identical settings and exposure times in paired experiments (**Fig 6B and 6C and S4 Fig**).

### Protein Expression and Purification

Plasmids encoding full-length HIZR-1(1–412) proteins with the wild-type (WT) or mutant sequence (G23E, S30L, or R63C) fused to an N-terminal 6XHis tag were transformed into BL21 *E*. *coli* cells. The empty vector pTrcHisA was transformed as a control. Cells were grown in Luria-Bertani media at 37°C, and expression was induced with 5 mM IPTG when the absorbance at 600 nm reached between 0.5–0.7. Expression was induced for 16–18 h at 16°C. Cells were then collected by centrifugation and suspended in 50 mM MOPS (pH 7.0). Cells were lysed by sonication in the presence of 0.16 mg/mL of lysozyme. Lysed cell material was pelleted by centrifugation and the supernatant was collected. HIZR-1 protein was purified from the supernatant using Clontech TALON Metal affinity resin. GST alone and the ligand-binding domains of HIZR-1 and DAF-12 fused to GST were expressed and harvested using the techniques described above; these proteins were purified using Genscript Glutathione Resin according to the manufacturer’s instructions and eluted with 10 mM L-glutathionine.

### Zinc Binding Assays

To analyze zinc binding to proteins, we used zinc-65 radionuclide (PerkinElmer, stock date 2/12/2015, specific activity 3.29 mCi/mg, concentration 5.90 mCi/mL, and radionuclidic purity 99.00%) and the purified proteins GST::HIZR-1(101–412 WT), GST::DAF-12(440–753 WT), and GST alone. Protein concentrations were quantified by Bradford assay. Equilibrium binding experiments were performed according to established guidelines [64]. Briefly, a constant amount of zinc-65 (0.01 μCi) was incubated with variable protein concentrations in 50 mM MOPS buffer with 10 mM L-glutathionine (pH 7.0) (**Fig 3A and 3B**). Based on the specific activity, we calculated that addition of 0.01 μCi zinc-65 results in a final concentration of zinc-65 of about 0.4 nM and a final concentration of total zinc (radioactive and nonradioactive) of about 1 μM. The half life of zinc-65 is 244 d, and it decays to copper-65. The number of decay half lives between production of the zinc-65 solution and binding experiments was less than one. Therefore, the amount of copper-65 was negligible compared to the amount of nonradioactive zinc in the source. Although purified proteins were eluted in buffer with no added zinc, we cannot exclude the possibility that the purified proteins contributed some nonradioactive zinc to the reaction mixture. Alternatively, a constant amount of protein (0.77 μM) was incubated with variable zinc concentrations (**S3 Fig**). Therefore, reactions consisted of (1) protein in 50 mM MOPS buffer with 10 mM L-glutathionine (pH 7.0) and (2) zinc dissolved in water. Reactions were allowed to equilibrate for 40 min, protein was vacuum blotted onto nitrocellulose membranes, membranes were briefly washed, and bound zinc-65 was quantified using a Beckman LS600 scintillation counter. At least two technical replicates were performed for each unique protein or zinc concentration. The dissociation constant was determined using GraphPad Prism software, assuming a 1:1 binding stoichiometry between zinc and the protein of interest.

To conduct the metal selectivity experiments, the binding interaction between a constant amount of GST::HIZR-1(101–412 WT) protein and zinc-65 was competed using no competitor or 500 μM of either nonradioactive zinc sulfate (ZnSO_4_), copper chloride (CuCl_2_), nickel chloride (NiCl_2_), or manganese chloride (MnCl_2_) (**Fig 3C**). Binding values were normalized by defining the binding interaction between the protein and zinc-65 with no nonradioactive competitor as maximal binding and setting that value equal to 1.0. Fraction maximal binding was calculated as the bound zinc-65 (CPM) for a given nonradioactive metal divided by the bound zinc-65 for no competitor (CPM).

### Electrophoretic Mobility Shift Assay (EMSA)

EMSAs were conducted using the Licor Odyssey EMSA Buffer Kit according to the manufacturer’s instructions. Images were captured with a Licor Odyssey Infrared Imager, and gel bands were quantified using Licor Image Studio software. To test the effect of the Zat-d (*am279*, *am280*, and *am287*) mutations on DNA-binding activity, a constant amount of full-length wild-type and mutant (G23E, S30L, and R63C) protein was incubated with a constant amount of labeled HZA oligonucleotide (**Fig 5A**). Protein concentrations were determined by Bradford assay and confirmed to be equivalent by western blot (**Fig 5B**). Proteins were visualized utilizing a Pierce 6XHis eptiope tag antibody with a Licor IRDye800 goat anti-mouse antibody. Blots were imaged with a Licor Odyssey Infrared Imager.

The dissociation constant was determined by incubating a constant concentration of full-length wild-type HIZR-1(1–412 WT) protein (pKW2) with variable concentrations of labeled HZA DNA (**Fig 5C**). The dissociation constant was calculated using GraphPad Prism software.

To conduct sequence specificity experiments, the binding interaction between a constant amount of HIZR-1(1–412 WT) protein and labeled HZA DNA was competed using variable concentrations of either unlabeled WT or mutant HZA DNA oligonucleotides (**Fig 5D**). The IRDye700 labeled HZA oligonucleotide was 5'- tgtgttatcaatcataaactagaacatgtctcgag-3'. The unlabeled oligonucleotides used were wild-type HZA, 5'-tgtgttatcaatcataaactagaacatgtctcgag-3' and Mutant HZA, 5'-tgtgttatcagaacatacaacattaatgtctcgag-3'. These DNA oligonucleotides were double stranded.

### Statistical Analysis

All data were analyzed utilizing the two-tailed Student’s *t* test of samples with unequal variance except for data in Fig 6D, which was analyzed by the Chi-squared test. When displayed, error bars indicate standard deviation. *p*-Values less than 0.05 were considered statistically significant. All the statistical comparisons in the current study consist of comparing two values made in different experiments performed in parallel. One type of comparison is wild-type animals compared to mutant animals, a pairwise comparison of two values determined in different experiments that were performed in parallel. A second type of comparison is the same genotype of animals compared in two distinct environmental conditions.

## Supporting Information

S1 Fig *hizr-1 *encodes a nuclear receptor similar to hepatocyte nuclear factor 4. The predicted amino acid sequence of HIZR-1 from *C*. *elegans *(Ce) is aligned with the predicted amino acid sequences of hepatocyte nuclear factor nuclear receptor from *Drosophila melanogaster *(Dm) and *Homo sapiens* (Hs). Amino acid numbers are shown on the right. The *C*. *elegans *DNA-binding domain is boxed in blue, and the ligand-binding domain is boxed in yellow. Amino acids affected by Zat-d mutations are boxed in red. The three recessive missense mutations affect highly conserved residues in the DNA-binding domain that are identical in worms, flies and humans. The two recessive nonsense mutations are predicted to generate truncated proteins that end at residue 86 (lacking the entire ligand-binding domain) and residue 226 (lacking part of the ligand-binding domain). The molecular analysis suggests that Zat-d mutations are strong loss-of-function or null alleles. The amino acid affected by the Zat-c missense mutation is boxed in green. The position is well conserved—an aspartic acid in worms and flies and a glutamic acid in humans. The semi-dominant *am285* missense mutation changes an acidic residue to a neutral asparagine. “*”, identical amino acids. “:”, amino acids share strongly similar properties. “.”, amino acids share weakly similar properties.
(TIF)

S2 Fig *ZK455*.*6/hizr-1 *is the gene affected by the Zat-d mutations. **(A)** mRNA was isolated from populations of wild-type (white) and *hizr-1(gk698405) *(gray) animals cultured with 0 or 200μM supplemental zinc, and *cdf-2 *transcript levels were analyzed by qPCR. mRNA levels are expressed in arbitrary units (A.U.) and were normalized to *rps-23*, a ribosomal protein gene that is not regulated by high zinc. The values were normalized by setting the value for wild-type animals at 0μM supplemental zinc equal to 1.0. Bars represent the average +/- S.D. (n = 3), (*, p < 0.05). In wild-type animals, *cdf-2 *transcript levels were increased significantly when cultured with 200μM supplemental zinc. Compared to wild-type animals, *hizr-1(gk698405) *mutant animals displayed significantly lower *cdf-2 *mRNA levels when cultured with 200μM supplemental zinc. **(B) **A diagram (not to scale) of the HIZR-1(1–412 WT)::GFP translational reporter construct containing the *hizr-1 *promoter (black line, 444 bp upstream of the ATG start codon) and the *hizr-1* coding region (open boxes are exons, and shading indicates untranslated regions) fused to the coding region of green fluorescence protein (green box). The *hizr-1* promoter contains a high zinc activated (HZA) enhancer element (orange box). This construct was injected into *hizr-1(am286lf)* mutant animals to generate transgenic animals with an extrachromosomal array. **(C)** mRNA was isolated from populations of *hizr-1(am286lf) *transgenic animals expressing HIZR-1(1–412 WT)::GFP (white) and their nontransgenic siblings that lost the extrachromosomal array (gray) cultured with 0 or 200μM supplemental zinc. Transgenic animals were identified by the Rol phenotype, whereas nontransgenic animals were identified by the nonRol phenotype. *cdf-2*, *ttm-1b*, and *mtl-1* transcript levels were analyzed by qPCR; mRNA levels were normalized to *rps-23*, a ribosomal protein gene that is not regulated by high zinc. Bars represent mRNA induction +/- S.D., calculated by dividing mRNA levels of animals cultured on 200μM supplemental zinc by those cultured on 0μM supplemental zinc (n = 3) (*, p < 0.05). High values indicate strong induction by high zinc. In nontransgenic *hizr-1(am286lf)* mutant animals, mRNA induction in high zinc was not substantial, demonstrating that the *am286* mutation abrogates high zinc activated transcription. By contrast, transgenic animals that expressed HIZR-1(1–412 WT)::GFP displayed a restoration of high zinc activated transcription, consistent with rescue of the mutant phenotype. The difference was statistically significant for *cdf-2 *and *ttm-1b*, whereas *mtl-1* displayed a trend that did not reach statistical significance with this sample size (p = 0.11).
(TIF)

S3 Fig Zinc directly bound the HIZR-1 ligand-binding domain. Glutathione S-transferase (GST) alone and the ligand-binding domain of HIZR-1 (residues 101–412) fused to GST were expressed in bacteria and partially purified by affinity chromatography. Increasing concentrations of radioactive zinc-65 were incubated with a fixed concentration of protein, and the amount of zinc-65 bound to protein was quantified by filter binding and scintillation counting. Values are the average +/- S.D. in counts per minute (CPM). GST::HIZR-1(101–412 WT) displayed saturable binding, and a non-linear regression was used to calculate a dissociation constant of 2.6 +/- 0.2 mM. X-ray crystallography studies indicate that GST binds one zinc molecule per protein [32]; our data are consistent with saturable, low-level zinc binding by GST alone.
(TIF)

S4 Fig *hizr-1 *was necessary for high zinc activated transcription mediated by the HZA enhancer. Transgenic animals containing the *3XHZApes-10p*::*gfp-nls* construct (basal *pes-10*+3XHZA) were either **(A,B)** *hizr-1(+)* or (**C,D**) *hizr-1(am286lf)*. Animals were cultured with **(A,C)** 0μM or **(B,D)** 200μM supplemental zinc. Representative images show the midbody region, and fluorescent puncta in panel B are intestinal nuclei containing nuclear localized GFP (representative nuclei are marked with arrowheads). Scale bars are approximately 25μm. Supplemental zinc caused the expression of nuclear localized GFP in *hizr-1(+)* animals, demonstrating that the promoter is activated by high dietary zinc. By contrast, *hizr-1(am286lf)* mutant animals did not display GFP expression in supplemental zinc, demonstrating that *hizr-1* is necessary for the high zinc activated transcription mediated by the HZA enhancer.
(TIF)

S1 Table *hizr-1* mutations. (TIF)

S1 Data Supporting data. (XLSX)

## Abbreviations

CDF: cation diffusion facilitator
CPM: counts per minute
DBD: DNA-binding domain
EMSA: Electrophoretic Mobility Shift Assay
GFP: green fluorescent protein
GST: glutathione-S-transferase
HZA: high zinc activation
LBD: ligand-binding domain
NR: Nuclear receptor
WT: wild type
ZRE: zinc-responsive element
