# Supplementary figures and images for: The Nuclear Receptor HIZR-1 Uses Zinc as a Ligand to Mediate Homeostasis in Response to High Zinc
Source: PLoS Biol. 2017 Jan 17;15(1):e2000094. doi: 10.1371/journal.pbio.2000094 (PMC5240932; doi:10.1371/journal.pbio.2000094)

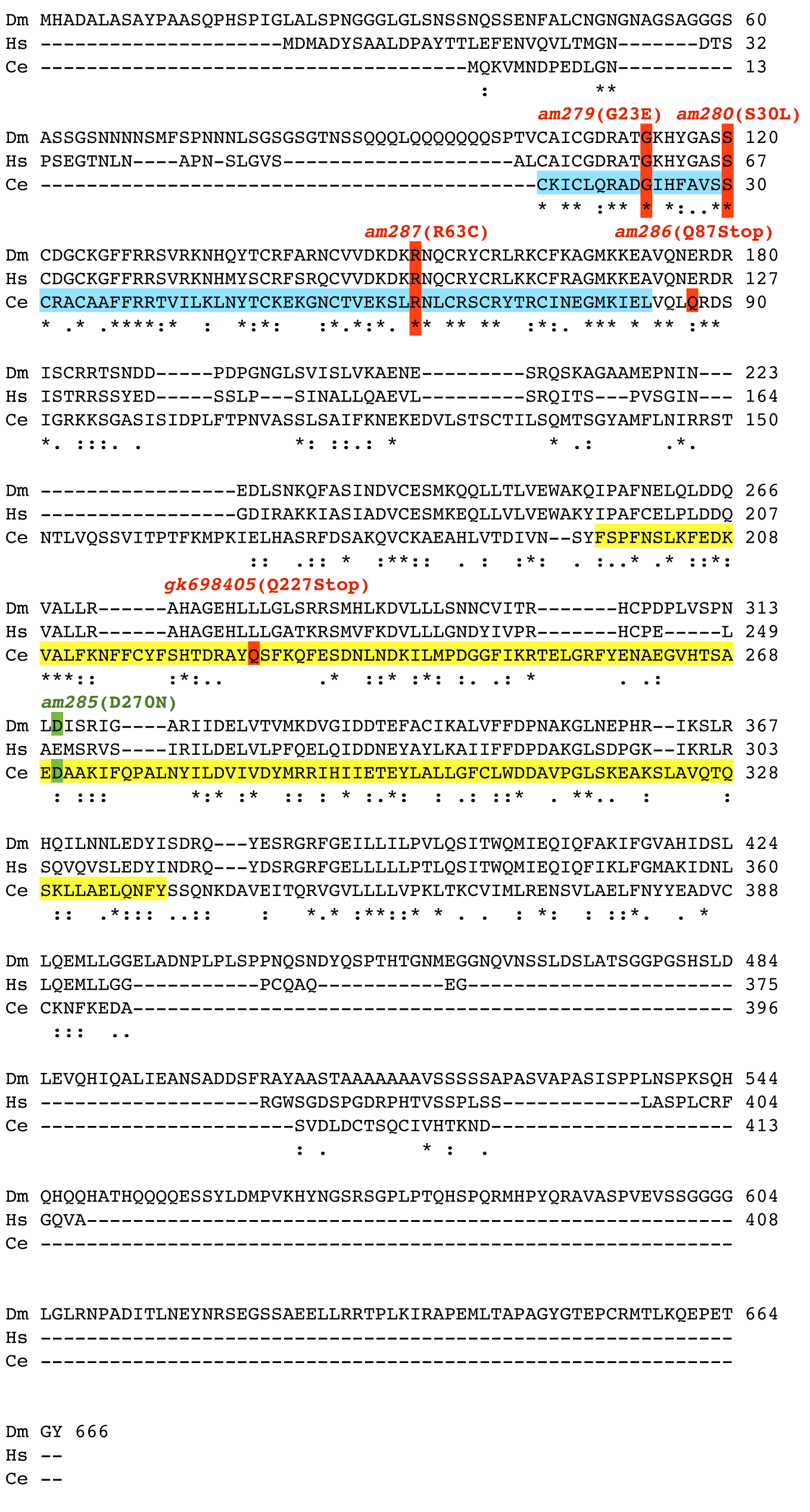

Supplement: S1 Fig — The predicted amino acid sequence of HIZR-1 from C. elegans (Ce) is aligned with the predicted amino acid sequences of hepatocyte nuclear factor nuclear receptor from Drosophila melanogaster (Dm) and Homo sapiens (Hs). Amino acid numbers are shown on the right. The C. elegans DNA-binding domain is boxed in blue, and the ligand-binding domain is boxed in yellow. Amino acids affected by Zat-d mutations are boxed in red. The three recessive missense mutations affect highly conserved residues in the DNA-binding domain that are identical in worms, flies and humans. The two recessive nonsense mutations are predicted to generate truncated proteins that end at residue 86 (lacking the entire ligand-binding domain) and residue 226 (lacking part of the ligand-binding domain). The molecular analysis suggests that Zat-d mutations are strong loss-of-function or null alleles. The amino acid affected by the Zat-c missense mutation is boxed in green. The position is well conserved—an aspartic acid in worms and flies and a glutamic acid in humans. The semi-dominant am285 missense mutation changes an acidic residue to a neutral asparagine. “*”, identical amino acids. “:”, amino acids share strongly similar properties. “.”, amino acids share weakly similar properties. (TIF) [file pbio.2000094.s001.tif]

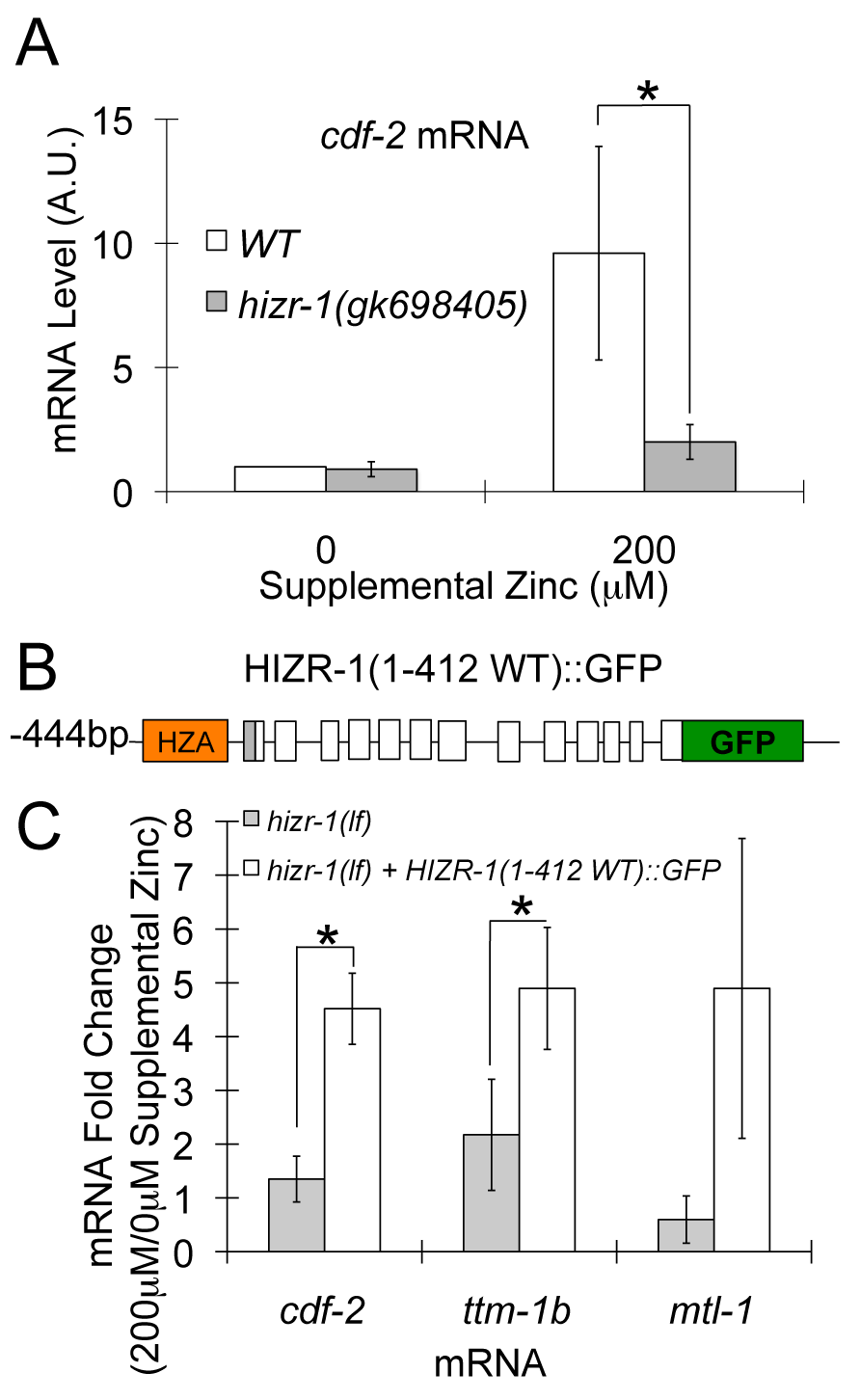

Supplement: S2 Fig — (A) mRNA was isolated from populations of wild-type (white) and hizr-1(gk698405) (gray) animals cultured with 0 or 200μM supplemental zinc, and cdf-2 transcript levels were analyzed by qPCR. mRNA levels are expressed in arbitrary units (A.U.) and were normalized to rps-23, a ribosomal protein gene that is not regulated by high zinc. The values were normalized by setting the value for wild-type animals at 0μM supplemental zinc equal to 1.0. Bars represent the average +/- S.D. (n = 3), (*, p < 0.05). In wild-type animals, cdf-2 transcript levels were increased significantly when cultured with 200μM supplemental zinc. Compared to wild-type animals, hizr-1(gk698405) mutant animals displayed significantly lower cdf-2 mRNA levels when cultured with 200μM supplemental zinc. (B) A diagram (not to scale) of the HIZR-1(1–412 WT)::GFP translational reporter construct containing the hizr-1 promoter (black line, 444 bp upstream of the ATG start codon) and the hizr-1 coding region (open boxes are exons, and shading indicates untranslated regions) fused to the coding region of green fluorescence protein (green box). The hizr-1 promoter contains a high zinc activated (HZA) enhancer element (orange box). This construct was injected into hizr-1(am286lf) mutant animals to generate transgenic animals with an extrachromosomal array. (C) mRNA was isolated from populations of hizr-1(am286lf) transgenic animals expressing HIZR-1(1–412 WT)::GFP (white) and their nontransgenic siblings that lost the extrachromosomal array (gray) cultured with 0 or 200μM supplemental zinc. Transgenic animals were identified by the Rol phenotype, whereas nontransgenic animals were identified by the nonRol phenotype. cdf-2, ttm-1b, and mtl-1 transcript levels were analyzed by qPCR; mRNA levels were normalized to rps-23, a ribosomal protein gene that is not regulated by high zinc. Bars represent mRNA induction +/- S.D., calculated by dividing mRNA levels of animals cultured on 200μM supplemental zinc by those cu [file pbio.2000094.s002.tif]

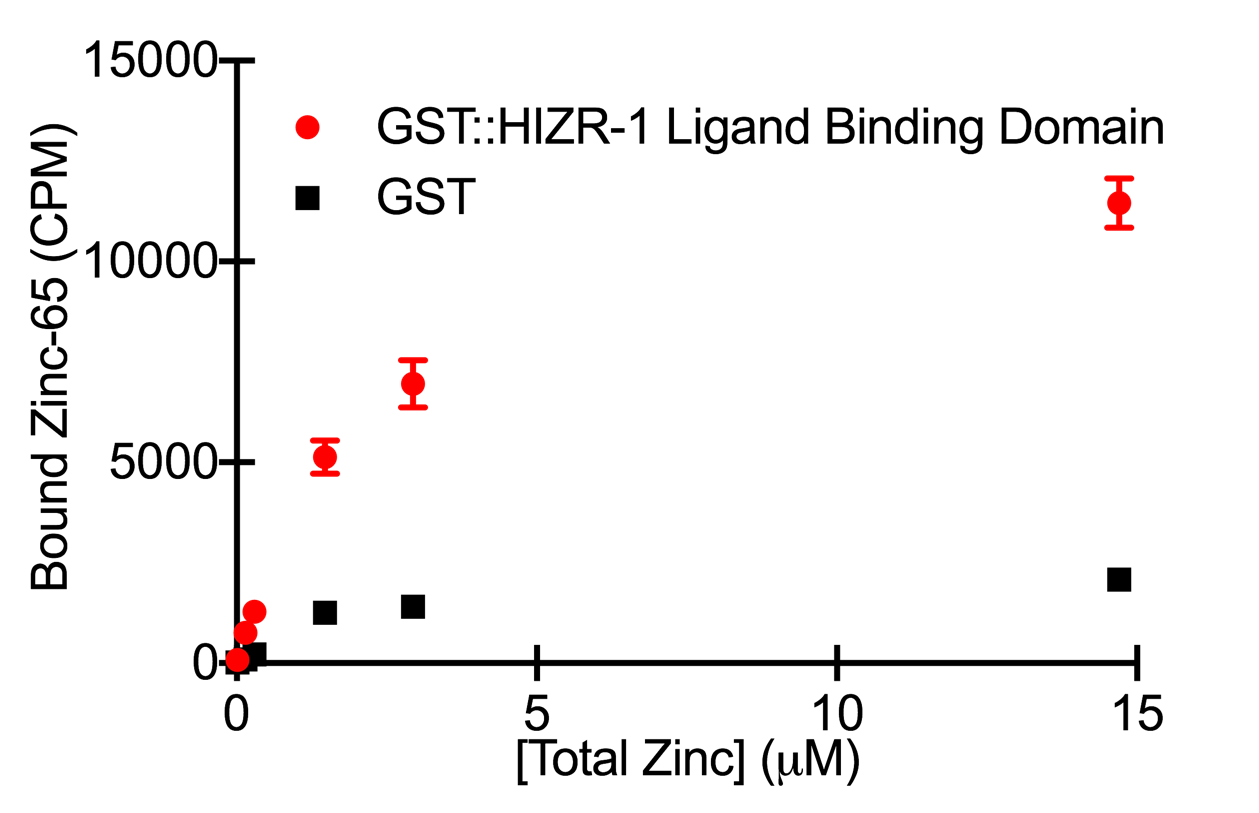

Supplement: S3 Fig — Glutathione S-transferase (GST) alone and the ligand-binding domain of HIZR-1 (residues 101–412) fused to GST were expressed in bacteria and partially purified by affinity chromatography. Increasing concentrations of radioactive zinc-65 were incubated with a fixed concentration of protein, and the amount of zinc-65 bound to protein was quantified by filter binding and scintillation counting. Values are the average +/- S.D. in counts per minute (CPM). GST::HIZR-1(101–412 WT) displayed saturable binding, and a non-linear regression was used to calculate a dissociation constant of 2.6 +/- 0.2 mM. X-ray crystallography studies indicate that GST binds one zinc molecule per protein [32]; our data are consistent with saturable, low-level zinc binding by GST alone. (TIF) [file pbio.2000094.s003.tif]

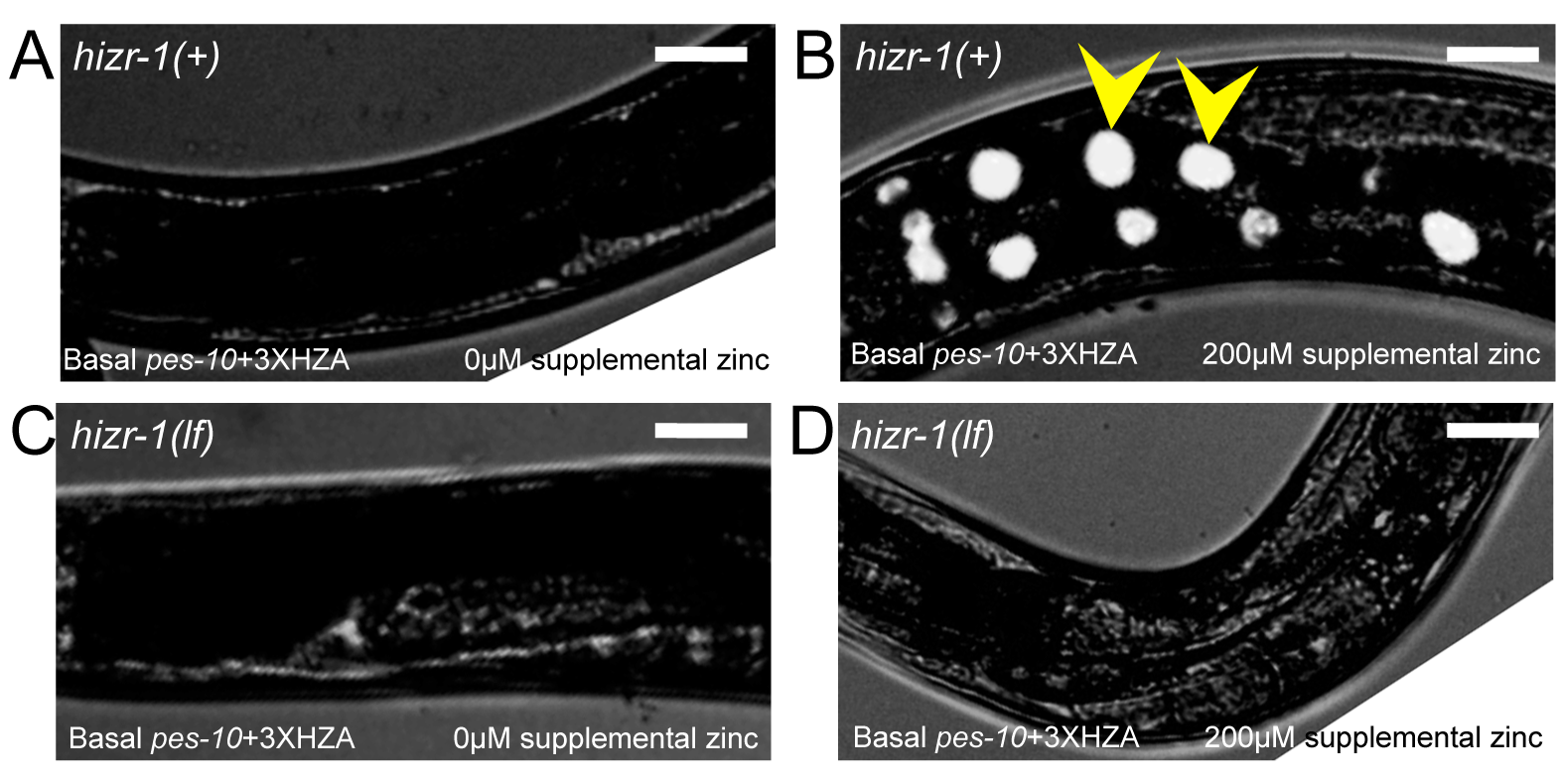

Supplement: S4 Fig — Transgenic animals containing the 3XHZApes-10p::gfp-nls construct (basal pes-10+3XHZA) were either (A,B) hizr-1(+) or (C,D) hizr-1(am286lf). Animals were cultured with (A,C) 0μM or (B,D) 200μM supplemental zinc. Representative images show the midbody region, and fluorescent puncta in panel B are intestinal nuclei containing nuclear localized GFP (representative nuclei are marked with arrowheads). Scale bars are approximately 25μm. Supplemental zinc caused the expression of nuclear localized GFP in hizr-1(+) animals, demonstrating that the promoter is activated by high dietary zinc. By contrast, hizr-1(am286lf) mutant animals did not display GFP expression in supplemental zinc, demonstrating that hizr-1 is necessary for the high zinc activated transcription mediated by the HZA enhancer. (TIF) [file pbio.2000094.s004.tif]

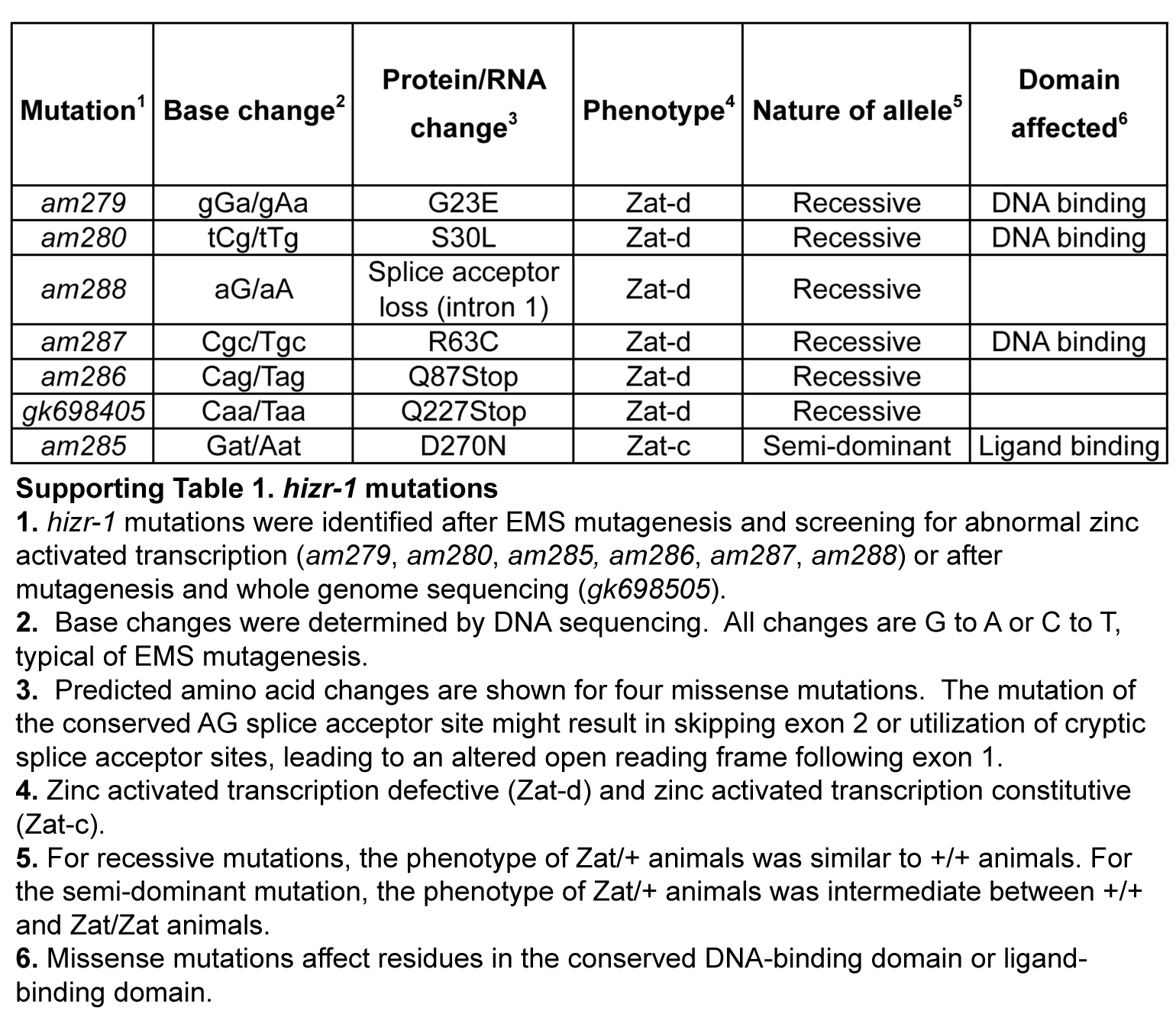

Supplement: S1 Table — (TIF) [file pbio.2000094.s005.tif]
